# Influence of water content and organic matter on the mechanical strength characteristics of municipal sewage sludge

**DOI:** 10.1371/journal.pone.0359715

**Published:** 2026-10-29

**Authors:** Ruimin Yang, Jiadong Ma, Zhenning Zhang, Jing Yin

**Affiliations:** 1 School of Architecture, Anhui Science and Technology University, Bengbu, Anhui Province, China; 2 Green Materials R&D Lab, Bengbu Architectural Design And Research Institute Group Co. Ltd, Bengbu, Anhui Province, China; IIT Roorkee: Indian Institute of Technology Roorkee, INDIA

## Abstract

Municipal sewage sludge typically has high moisture and organic matter contents, which exert a significant influence on mechanical strength, dewatering and resource recovery. Organic soil specimens with varying water contents were prepared by mixing raw sewage sludge (RSS) with dry soil powder (DSP), humus, and quartz sand tailings (QST). Unconfined compressive strength (UCS) tests were conducted to investigate the effect of water content on the UCS. Results show that the UCS decreases with normalized water content (*w*₀/*w*_L_) following a power-law relationship, and a quantitative formula is established. Specimens with different organic contents were fabricated by blending DSP into RSS. Unconsolidated undrained triaxial tests were performed to assess strength evolution with organic content. For organic high-liquid-limit silt, the angle of internal friction changes slightly with organic content (*w_u_*), maintaining a low value of 1–3°. Cohesion decreases gradually when *w_u_* < 17%, drops sharply when 17% < *w*_u_ < 21%, and stabilizes at around 6 kPa when *w*_u_ > 21%. An organic content of 17–21% serves as a possible transition interval for sludge structure and strength degradation. Mechanism analysis reveals that the contribution of organic matter to soil strength is predominantly governed by cementation, with negligible influence on the internal friction angle. The reduction in cohesion is primarily attributed to the decrease in dry unit weight, degradation of mineral skeleton, a predominance of the soft organic matrix, and increased plastic deformation.

## Introduction

Municipal sewage sludge, an inevitable byproduct of urban wastewater treatment processes, presents significant challenges for sustainable management due to its high-water content and complex organic composition [1,2]. The steadily rising volume of sludge produced worldwide, driven by population growth and industrialization, necessitates innovative treatment and disposal strategies [3,4]. Traditional disposal methods such as landfilling and incineration are increasingly confronted with environmental concerns, regulatory restrictions, and economic burdens [5,6]. Consequently, there is an urgent need to explore alternative, more sustainable approaches, including resource utilization and material recycling [7−9]. A solid understanding of the mechanical and geotechnical properties of sewage sludge is necessary for its efficient dewatering, stabilization, and possible reuse in various fields, particularly in building and agriculture [10−12].

The inherent characteristics of municipal sewage sludge, particularly its water content and organic matter composition, profoundly influence its strength properties and overall behavior [13−15]. The compressible properties of sludge and shear strength are strongly affected by its high-water content, which typically ranges from 0.13 to 1.48 times its liquid limit [14]. Shear strength exhibits a strong negative correlation with sludge moisture content [16,17]. As moisture content rises, the effective interparticle stress within the sludge diminishes, along with an increase in pore water pressure—ultimately resulting in a marked decrease in shear strength [16]. O’Kelly (2013) examined the link between undrained shear strength and moisture content in sewage sludge [18]. His work also addressed the factors governing this relationship and concluded that moisture content plays a decisive role in determining the undrained shear strength of sludge. Furthermore, the organic composition of municipal sewage sludge significantly influences its compression properties, which in turn influences its appropriateness for disposal in landfills and recovery of resources [13]. High levels of organic components, particularly extracellular polymeric substances (EPS), hinder dewatering processes and contribute to the sludge’s complex rheological behavior [19,20]. The gel-like structure of EPS traps water within sludge flocs, posing major challenges to achieving efficient deep dewatering [21]. This high-water content of sludge caused by its high organic content also significantly affects the unconfined compressive strength of sludge-based materials, which is a key parameter required for the application of these materials in fields such as landfill cover materials or construction aggregates [22−24]. In solidified sludge, unconfined compressive strength decreases with increasing organic matter content [23,25]. The strength development of cement-stabilized clay is impacted by larger organic matter contents because they raise the water content in cemented clay [25,26]. Cao et al. (2022) provided further evidence that organic matter negatively affects strength, shedding light on the complexity of the underlying mechanism [23].

Although these studies have yielded substantial progress, significant challenges remain in fully understanding how organic matter affects sludge strength and in optimizing its mechanical properties. The macroscopic mechanical behavior of sludge under different moisture contents continues to be a central research focus [15], and while qualitative or site‑specific trends have been reported, no reliable quantitative relationship has yet been established to describe how the strength of different sludge types evolves with moisture content across a wide range of normalized water contents. While O’Kelly (2013) has established seminal quantitative relationships between undrained shear strength and water content for certain organic soils, these models were primarily developed based on clay-dominant sludge [18]. Due to the significant differences in fabric and particle morphology between clayey and silty organic sludges, the applicability of these existing models to the widely distributed organic high-plasticity silt (MH) in China remains uncertain. Therefore, the objective of this study is not to establish a totally new relationship from scratch, but to calibrate, validate, and extend the existing framework to a broader spectrum of sludge types, using a comprehensive database pooled from multiple regional studies. The long-term mechanical performance and durability of sludge-modified materials, which are highly dependent on organic matter, still require systematic and in-depth investigation [22,27]. Moreover, the specific ways in which organic matter in sludge affects its strength, how organic content governs shear strength parameters, and the underlying mechanisms behind these effects remain poorly understood. In particular, previous studies have often treated organic matter as a lumped factor without distinguishing its differential impacts on cohesion versus internal friction angle, nor have they identified any threshold organic content beyond which strength collapses dramatically—information that is critically needed for selecting pretreatment methods and assessing sludge suitability for various reuses.

In this study, sludge samples with different moisture and organic contents were tested using triaxial compression and unconfined compressive strength experiments. After comprehensively analyzing the effect of moisture content on sludge strength characteristics, a power-law model was developed to quantitatively characterize the relationship between moisture content and sludge strength. Additionally, the effects of organic matter on the mechanical properties of municipal sewage sludge were examined, and the variation patterns of shear strength with changing organic content were identified. Specifically, the main contributions of this work are threefold: (i) we propose a quantitative power‑law formula linking normalized water content (*w*_₀_/*w*_L_) to UCS, which provides a practical predictive tool for engineers dealing with sludge from different sources; (ii) we identify, for the first time, a critical organic content window of 17%–21% that marks the transition from gradual to drastic strength loss, offering a possible transition interval for sludge classification and process design; (iii) we decouple the effects of organic matter on cohesion and internal friction angle, revealing that organic matter acts primarily by weakening cementation‑induced cohesion rather than by altering frictional resistance, and we attribute the cohesion decay to the coupled effects of reduced dry density, skeleton deterioration, and matrix softening. This work provides a valuable reference for the efficient management and resource utilization of sludge, laying a theoretical basis and practical guidance for solving related engineering problems.

## Materials and methods

Raw sewage sludge (RSS) for the present study was collected from a wastewater treatment plant located in Chuzhou City, Anhui Province, China. Derived from domestic sewage, the sludge was treated via “thickening‑conditioning‑high‑pressure plate‑and‑frame dewatering”. The water content of the discharged sludge cake was less than 80%. After temporary storage onsite, the sludge was transported off‑site for disposal. Its pH was approximately 6.5, volatile solids (dry basis) around 45%, and ash content about 50%. The inorganic minerals were dominated by silicates such as quartz and feldspar, together with montmorillonite and calcite.

This RSS material was then labeled as Specimen A to investigate the impact of initial moisture content on sludge strength. Subsequently, Specimens B, C, and D were prepared by blending the RSS with 20% dry soil powder (DSP), 20% humus and 20% quartz sand tailings (QST), respectively. The DSP employed in the test was derived from excavated spoil generated during foundation pit excavation in Bengbu City, Anhui Province, China, which was air-dried and crushed prior to use. Table 1 summarizes the physical properties of Specimens A–D, and Fig 1 presents their particle size distribution curves. The particle size distribution curves for DSP, humus, and QST are also shown in Fig 1. For the DSP, the contents of clay (< 2 μm), silt (2 − 60 μm), and sand (60 − 2000 μm) were measured as 6.6%, 89.8%, and 3.6%, respectively. With an organic content of 1.68%, DSP is classified as inorganic silt. In this section, we need to provide information regarding the permits obtained for this work. However, no site access permits were required for this study, since field work was carried out in open public areas outside protected ecological zones. As our non-intrusive investigative activities caused no harm to the surrounding environment, no administrative approval was applicable.

**Table 1:** Physical indicators of sludge specimens A, B, C and D.

Specimens	Organic content, *w*_u_(%)	Initial water content, *w*_0_(%)	Unit weight, *γ* (kN/m^3^)	Dry unit weight, γ_d_ (kN/m^3^)	Liquid limit, *w*_L_ (%)	Plastic limit, *w*_p_ (%)	*w*_0_/*w*_L_
A: RSS	26.06	62.25	11.670	7.192	249.00	101.29	0.25
		89.64	12.740	6.719			0.36
		117.03	12.160	5.604			0.47
		141.93	12.804	5.292			0.57
		169.32	12.464	4.628			0.68
B: RSS + 20% DSP	17.60	58.50	16.019	10.107	150.00	55.50	0.39
		72.00	15.111	8.785			0.48
		87.00	13.545	7.243			0.58
		100.50	13.615	6.791			0.67
		117.00	13.651	6.291			0.78
C: RSS + 20% Humus	45.50	44.82	13.957	9.637	166.00	74.87	0.27
		73.04	12.780	7.386			0.44
		87.98	13.772	7.326			0.53
		101.26	13.388	6.652			0.61
		117.86	11.759	5.398			0.71
D: RSS + 20% QST	17.08	46.34	15.981	10.92	136.30	80.58	0.34
		68.15	14.131	8.404			0.50
		87.23	14.249	7.610			0.64
		100.86	12.897	6.421			0.74
		119.94	13.390	6.088			0.88

**Table 2:** Physical indicators of sludge specimens (#1, #2, #3, #4, #5 and #6).

Specimens	Dosage of DSP, *λ* (%)	Organic content, *w*_u_(%)	Initial water content, *w*_0_(%)	Unit weight, *γ* (kN/m^3^)	Liquid limit, *w*_L_ (%)	Plastic limit, *w*_p_ (%)	*w*_0_/*w*_L_
#1(RSS)	0	27.03	203.98	12.788	217.00	105.51	0.94
#2	5	23.38	153.69	13.280	185.45	87.85	0.83
#3	10	20.67	132.66	13.900	150.23	74.74	0.88
#4	20	16.78	96.89	14.300	129.56	55.92	0.75
#5	40	11.63	65.76	15.450	85.45	38.56	0.77
#6	90	6.91	35.94	18.350	45.42	28.45	0.79

**Fig 1:**
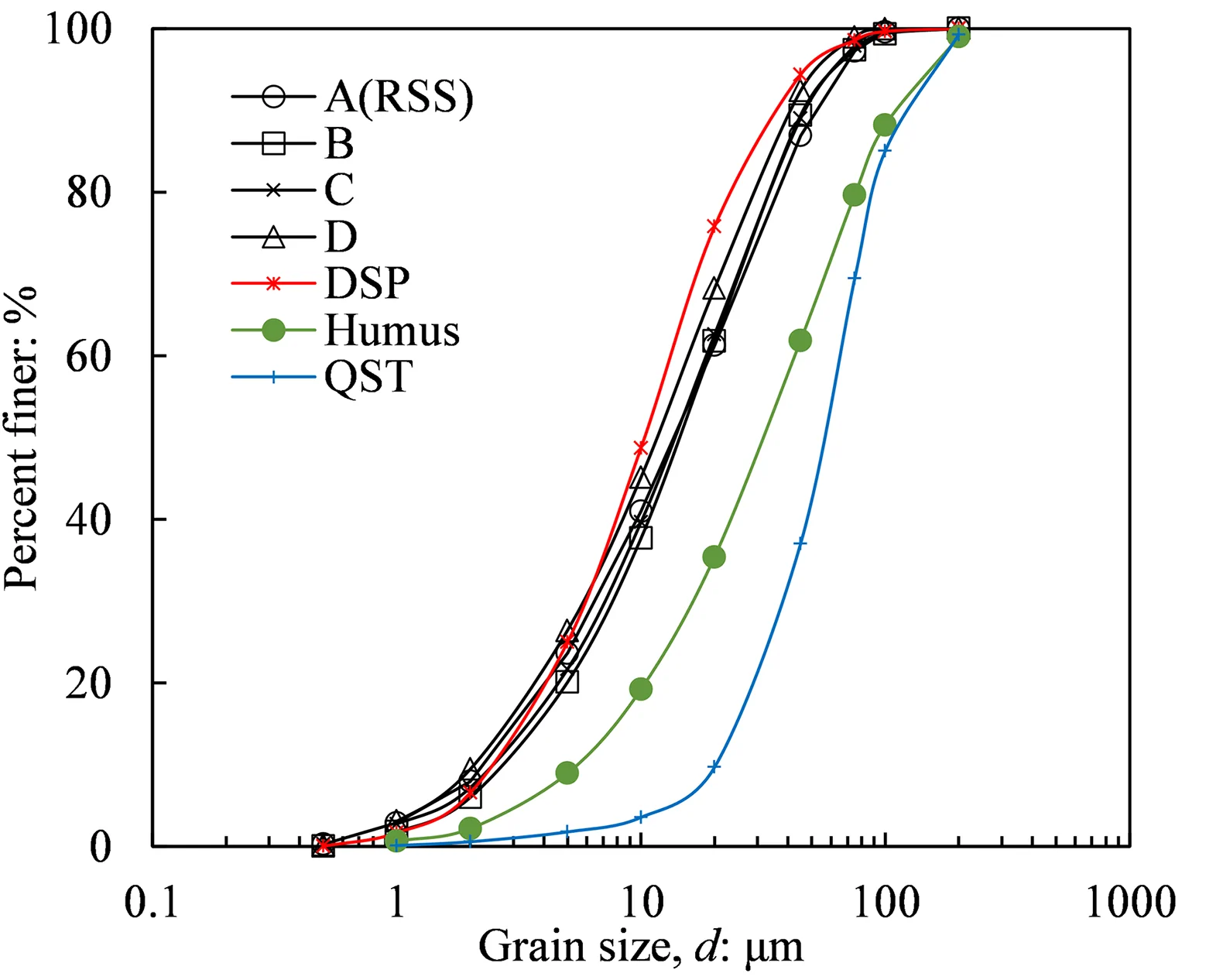
Particle size analysis curves of sludge specimens A, B, C and D, DSP, humus and QST.

**Fig 2:**
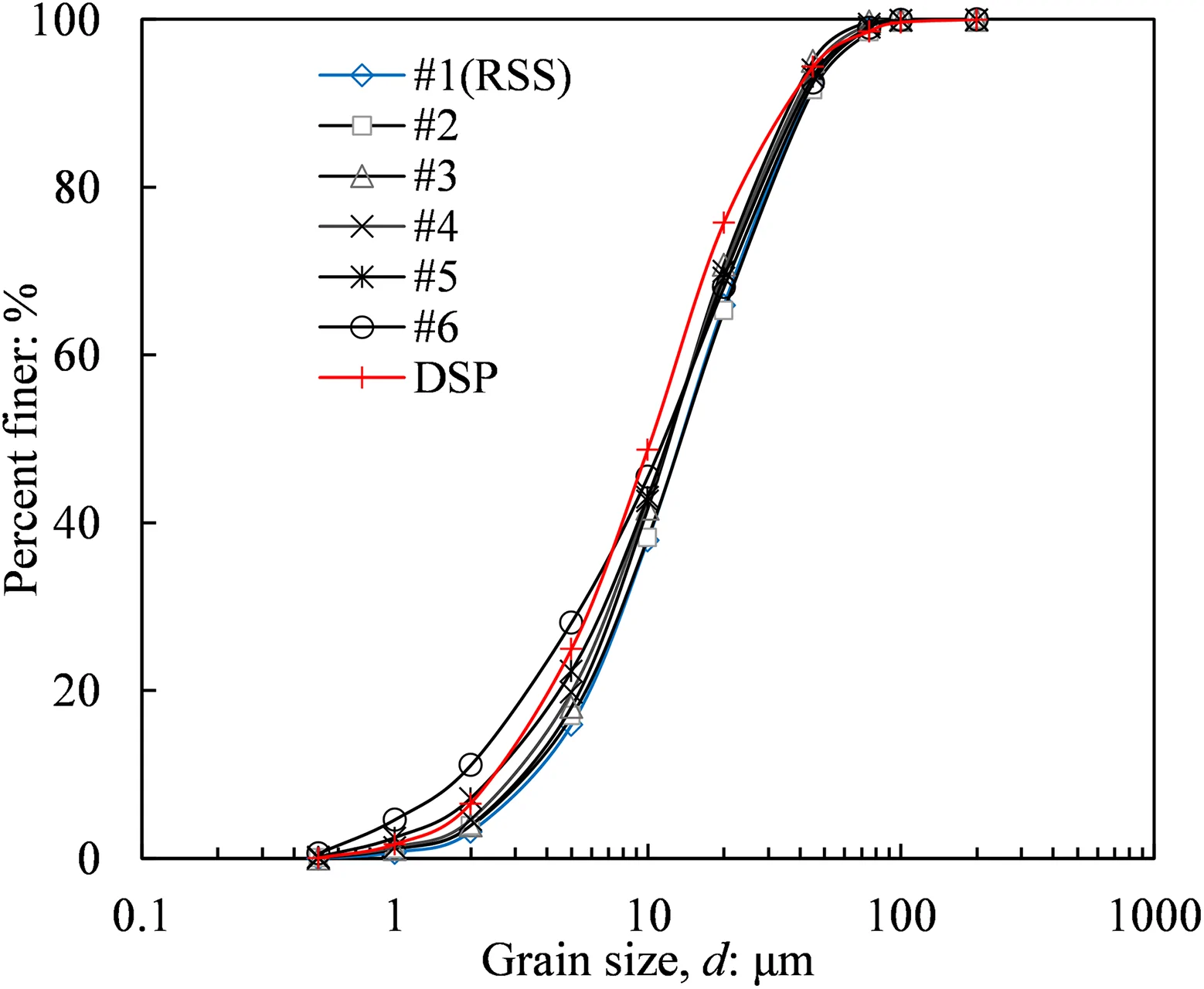
Particle size analysis curves of sludge specimens (#1, #2, #3, #4, #5 and #6).

The QST utilized in the study came from a tailings pond next to a glass factory in Chuzhou City, Anhui Province, China. As shown in Fig 1, the clay (< 2 μm), silt (2–60 μm), and sand (60–2000 μm) fractions of QST were measured as 0.6%, 56.0%, and 43.4%, respectively, with an organic content of 0.38%. Potassium humate, a humic acid product with a purity of over 95%, was purchased and used as the humus material in the tests. Similarly, the particle size distribution of humus was determined as 2.2% clay (< 2 μm), 75.8% silt (2–60 μm), and 22.0% sand (60–2000 μm) (see Fig 1), with an organic content of 78.92%. Table 1 summarizes the physical properties of Specimens A, B, C, and D, including organic content, initial water content, unit weight, dry unit weight, liquid limit, and plastic limit. To explore the contribution of moisture content to the strength of the four organic soils (Specimens A–D), each soil was prepared at a series of different moisture contents (see Table 1), followed by unconfined compressive strength (UCS) tests.

To investigate the effect of organic content on sludge strength, DSP was added to RSS at mass ratios of 0, 5, 10, 20, 40, and 90% to prepare organic soil specimens with graded organic contents, denoted as Specimens #1 to #6, respectively. To minimize the role in moisture content, moisture content was normalized by controlling the ratio *w*₀/*w*_L_ within the range of 0.75–0.94. The physical indices of Specimens #1–#6 are listed in Table 2, and their particle size distribution curves are shown in Fig 2.

Potassium humate was selected as the model organic matter in this experiment. Geologically originated, it features an aromatic backbone and abundant carboxyl and phenolic hydroxyl groups, and exhibits alkaline properties. By contrast, native extracellular polymeric substances (EPS) in sludge are biologically derived, mainly consist of proteins and polysaccharides, and are nearly neutral. There are fundamental differences between potassium humate and native sludge EPS in terms of origin, chemical composition and physicochemical properties, which may lead to partial overestimation of the cementation enhancement effect of organic matter and the evolution law of microstructures in the experiment. Accordingly, the conclusions drawn from this study are primarily applicable to organic‑matter systems dominated by humic‑substance components. Caution should be exercised when extrapolating these findings to systems dominated by proteins and polysaccharides.

Moisture content of the sludge was determined using the drying oven method [28,29]: a 100 g sample was dried at 65 °C for 48 h. Unit weight was measured using the ring knife method [30]. During sludge preparation, compaction energy was kept consistent by controlling hammer weight, drop height, and compaction frequency. Using the measured values of moisture content and unit weight, the dry unit weight was subsequently determined. The liquid limit is defined as the water content at which a cone penetrates to a depth of 17 mm, as measured by the cone penetrometer method [30]. The plastic limit was established using the roll‑out method [31]. Organic content was determined referring to the following method [28,32,33]: the specimen was crushed to guarantee even particle dispersion, dehydrated for 48 hours at 65 °C, and then ignited at 550 °C to measure the loss on ignition. Sludge samples were subjected to UCS experiments and unconsolidated undrained triaxial tests in accordance with relevant standards [30,34,35]. To prepare homogeneous specimens, the required quantities of dry sludge powder (DSP), quartz sand (QST), and potassium humate were first thoroughly dry‑mixed in a mechanical blender at low speed for 5 min. De‑aired distilled water (pre‑boiled and cooled to room temperature) was then gradually added while mixing continued for another 10 min, after which the mixture was manually kneaded until a uniform paste was obtained. The paste was compacted into cylindrical moulds in three equal layers, with each layer lightly tamped to ensure consistency. After demoulding, each specimen was wrapped in plastic film and stored in a sealed humid container at room temperature for 24 h prior to testing, allowing moisture to equilibrate throughout the sample. For the triaxial tests, specimens were saturated using the vacuum saturation method: they were placed in a vacuum chamber, evacuated for 30 min, and then flooded with de‑aired water while maintaining vacuum for an additional 24 h. Saturation was confirmed by measuring the B‑value, with values exceeding 0.95 accepted as fully saturated. All specimens were 80 mm in height and 39.1 mm in diameter, with a shear rate of 0.5% per minute. The strength tests were performed at a room temperature of 27 °C. The peak stress, or the stress at a vertical strain of 15% if no distinct peak occurred, was adopted as the failure stress. A series of triaxial compression tests were carried out under confining pressures of 100, 200, and 300 kPa.

### Physical properties

#### Physical properties of specimens A, B, C and D.

Fig 3 shows the variation in dry unit weight (*γ*_d_) with normalized water content (*w*_0_/*w*_L_) for the four samples (A, B, C and D). For all specimens, *γ*_d_ declines as *w*_0_/*w*_L_ rises. The positions of samples A, B, C, and D on the Casagrande plasticity chart are presented in Fig 4. They are all located below the A-line and to the right of the B-line, and are classified as organic high-liquid-limit silt. Correlation analysis shows that *γ*_d_ decreases exponentially with *w*_0_/*w*_L_, with high correlation and statistical significance. The regression equations describing the relationship between *γ*_d_ and *w*_0_/*w*_L_ for the four organic soil samples A, B, C, D are presented in Table 3. As shown, four samples exhibit a strong and statistically significant correlation between *γ*_d_ and *w*_0_/*w*_L_. The absolute values of correlation coefficients are all above 0.97, with p-values less than 0.01 for all samples. Residual analysis indicated that the residuals of dry unit weight and normalized moisture content followed a normal distribution (Shapiro-Wilk, *p* > 0.05) and were statistically independent (Durbin-Watson, 1.5 < *d* < 2.5), satisfying all regression assumptions. Given the small sample size (*n* = 5), the generalizability of conclusions from this study requires further validation using larger datasets. Nevertheless, residual diagnostics confirm that the regression relationship derived from the present data is statistically reliable.

**Table 3:** Correlation equations of dry unit weight (*γ*_d_) versus normalized water content (*w*_0_/*w*_L_) for specimens A, B, C and D.

Specimens	Correlation equations	Correlation Analysis and Significance Description
A(RSS)	*γ*_d_ = 9.3503 e^−1.007(*w*0/*w*L)^	*n* = 5, *R*=−0.987, *p*-value <0.001, *SE*r = 0.093, 95% *CI* [−0.999, −0.811], Shapiro-Wilk *p* = 0.332, Durbin-Watson *d* = 1.93, *R*² = 0.973, *RMSE* (kN/m³)=0.154
B	*γ*_d_ = 15.698 e^−1.215(*w*0/*w*L)^	*n* = 5, *R*=−0.977, *p*-value = 0.004, *SE*r = 0.123, 95% *CI* [−0.999, −0.687], Shapiro-Wilk *p* = 0.779, Durbin-Watson *d* = 1.62, *R*² = 0.958, *RMSE* (kN/m³)=0.289
C	*γ*_d_ = 13.308 e^−1.213(*w*0/*w*L)^	*n* = 5, *R*=−0.974, *p*-value = 0.005, *SE*r = 0.131, 95% *CI* [−0.998, −0.650], Shapiro-Wilk *p* = 0.679, Durbin-Watson *d* = 1.92, *R*² = 0.955, *RMSE* (kN/m³)=0.293
D	*γ*_d_ = 15.311 e^−1.102(*w*0/*w*L)^	*n* = 5, *R*=−0.982, *p*-value = 0.003, *SE*r = 0.109, 95% *CI* [−0.999, −0.746], Shapiro-Wilk *p* = 0.628, Durbin-Watson *d* = 2.25, *R*² = 0.969, *RMSE* (kN/m³)=0.306

Note: *n* – number of samples; *R* – correlation coefficient; *p*-value – significance; *SE*_r_ – standard error; *CI* [] – 95% confidence interval.

**Fig 3:**
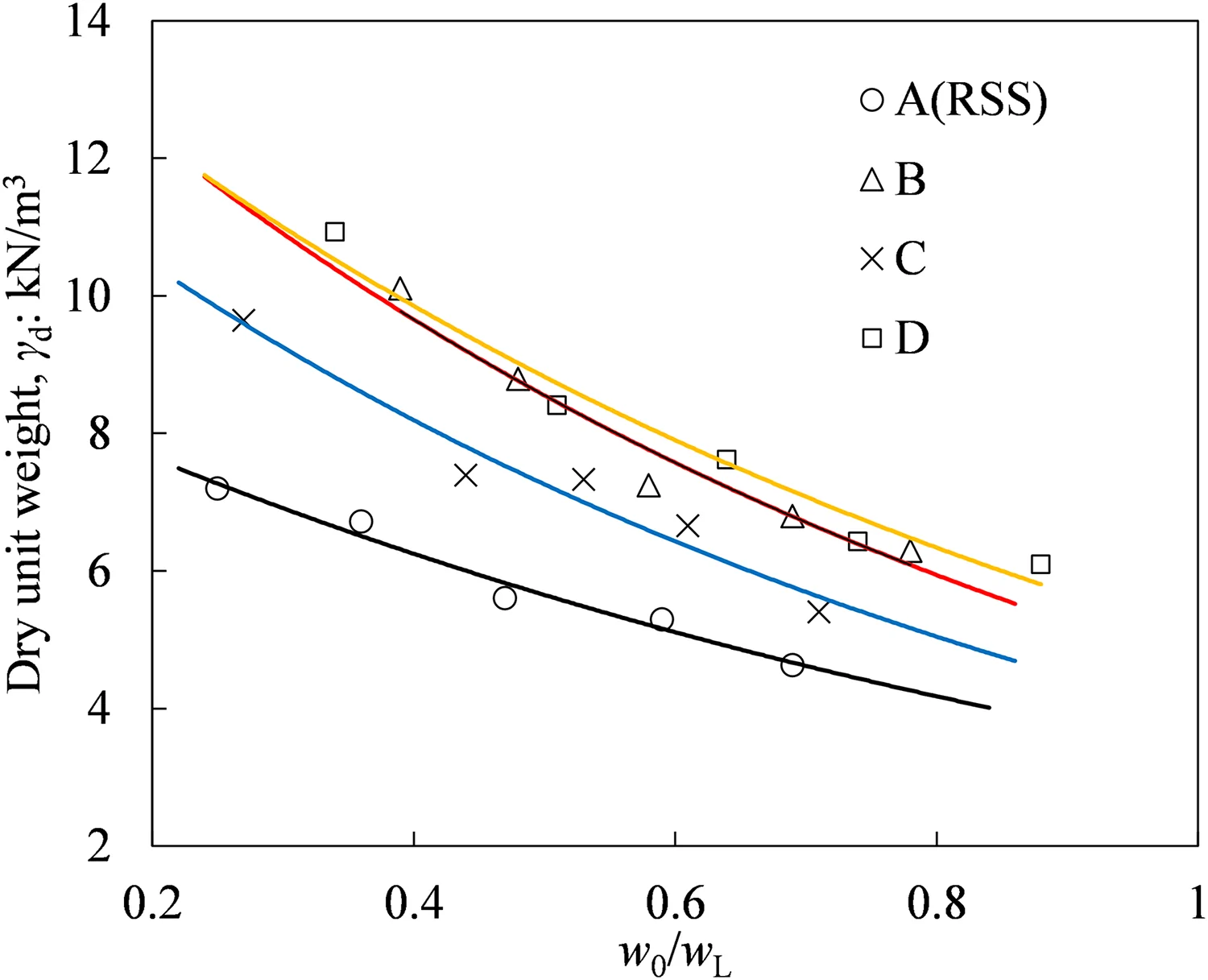
Dry unit weight (*γ*_d_) versus normalized water content (*w*_0_/*w*_L_) for four sludge specimens.

**Fig 4:**
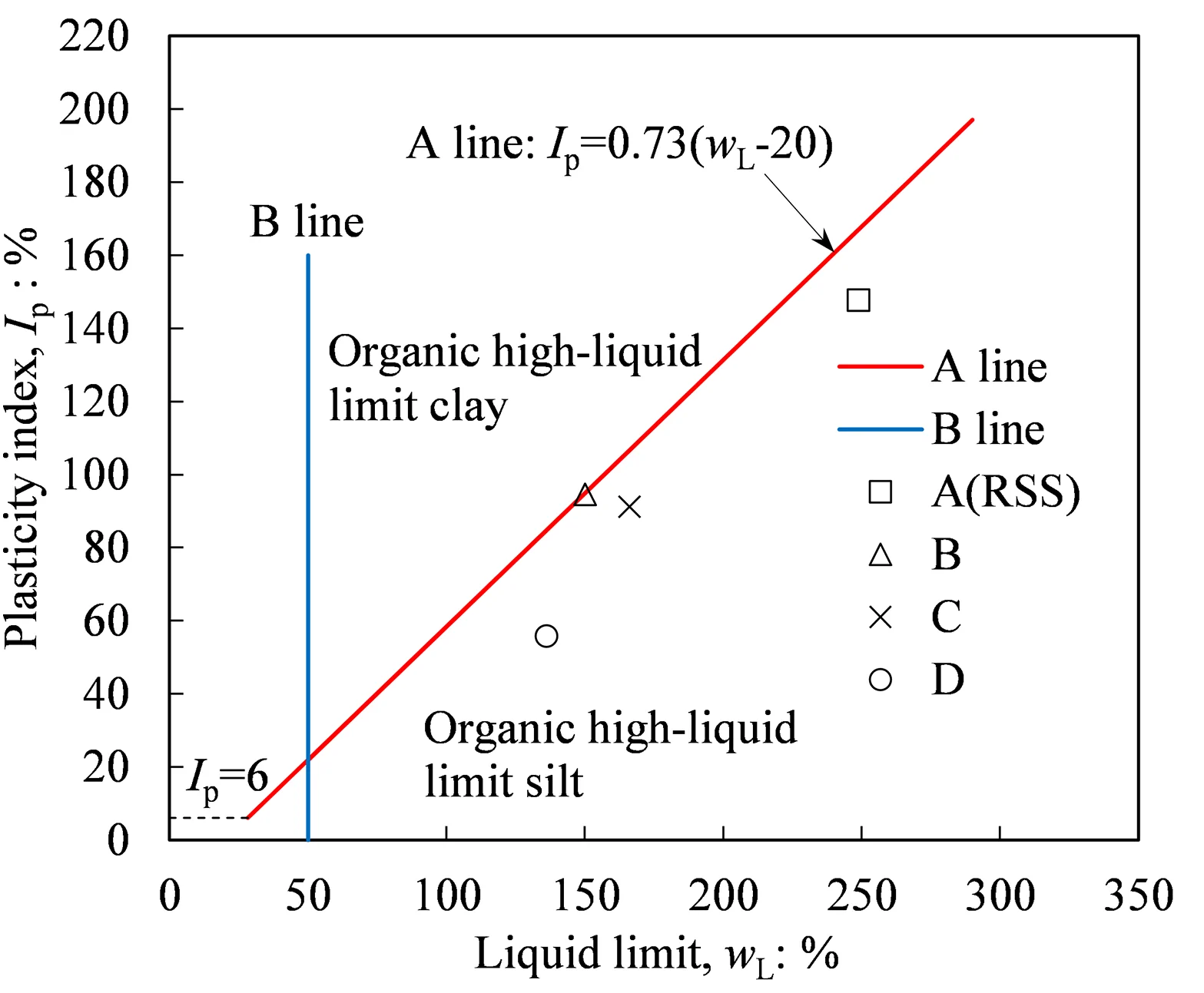
Distribution of sludge specimens A, B, C and D on the Casagrande plasticity chart.

As shown in Fig 3 and Table 3, when organic content exceeds 15%, the dry unit weight does not follow the bell-shaped curve typical of inorganic clays—rising to a peak and then falling. Instead, it decreases roughly exponentially with increasing *w*_0_/*w*_L_. This difference stems from the low density, high water retention, and elasticity of organic matter, which bring the soil near saturation well below the liquid limit. Further wetting builds up pore pressure rapidly, and compaction energy is absorbed by water rather than transmitted to soil particles, causing the dry unit weight to drop steadily. For such highly organic soils, the traditional optimum water content therefore loses practical meaning; field control should emphasize limiting water content rather than chasing a maximum dry density. This trend agrees with published observations on the compressibility and compaction behaviour of organic soils.

### Physical properties of specimens #1, #2, #3, #4, #5 and #6

Fig 5 illustrates the variation in sludge organic content with DSP dosage. It is clear that organic content decreases exponentially as DSP dosage increases. Fig 6 shows the relationship between unit weight, dry unit weight and organic content. It reveals that both unit weight and dry unit weight decrease as organic content increases. Correlation analysis indicates that unit weight decays as a logarithmic function of organic content (Eq 1), whereas dry unit weight decays exponentially (Eq 2). The regression equation for unit weight as a function of organic content is presented as follows:

**Fig 5:**
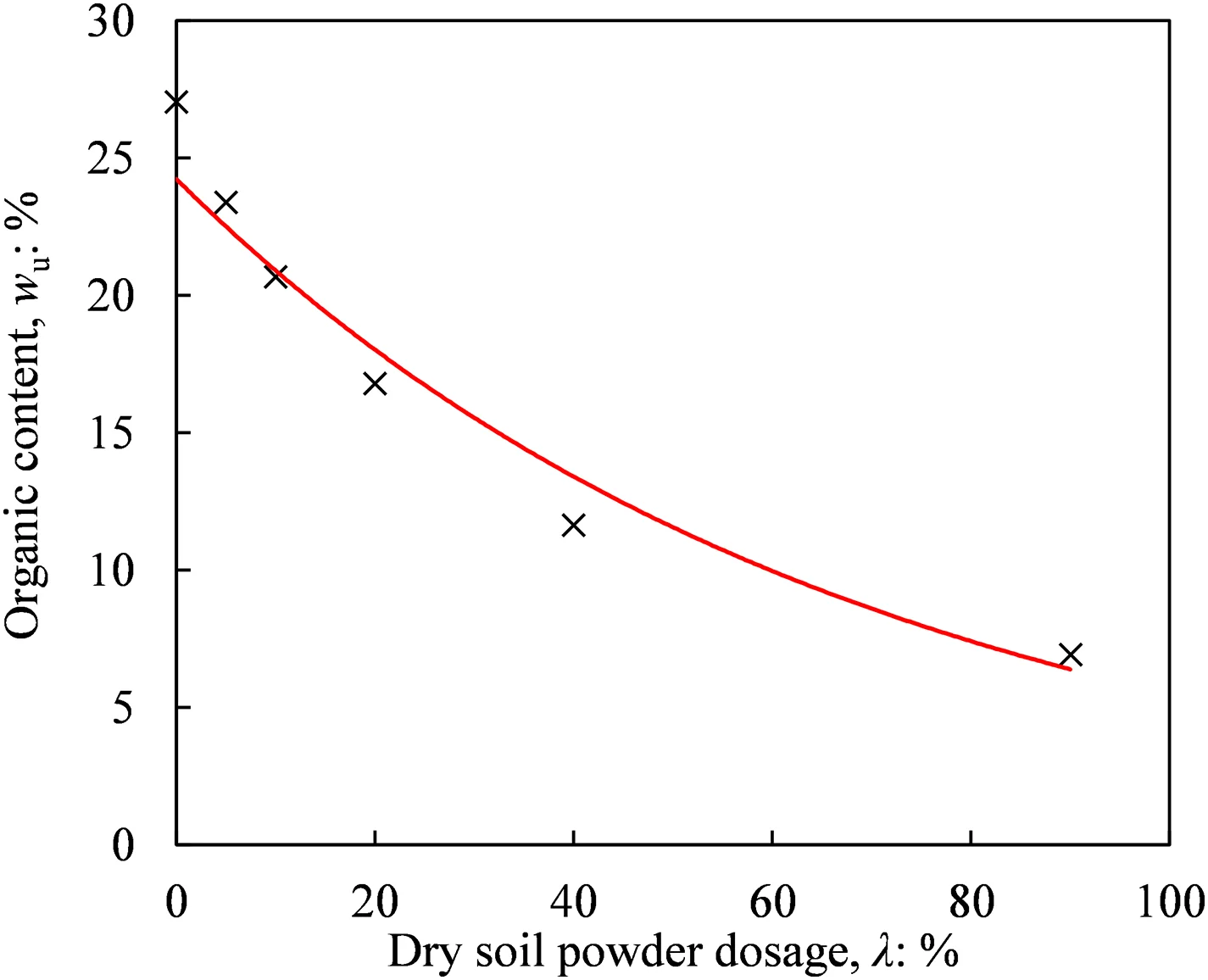
Relationship between organic content (*w*_u_) and dosage of DSP (*λ*).

**Fig 6:**
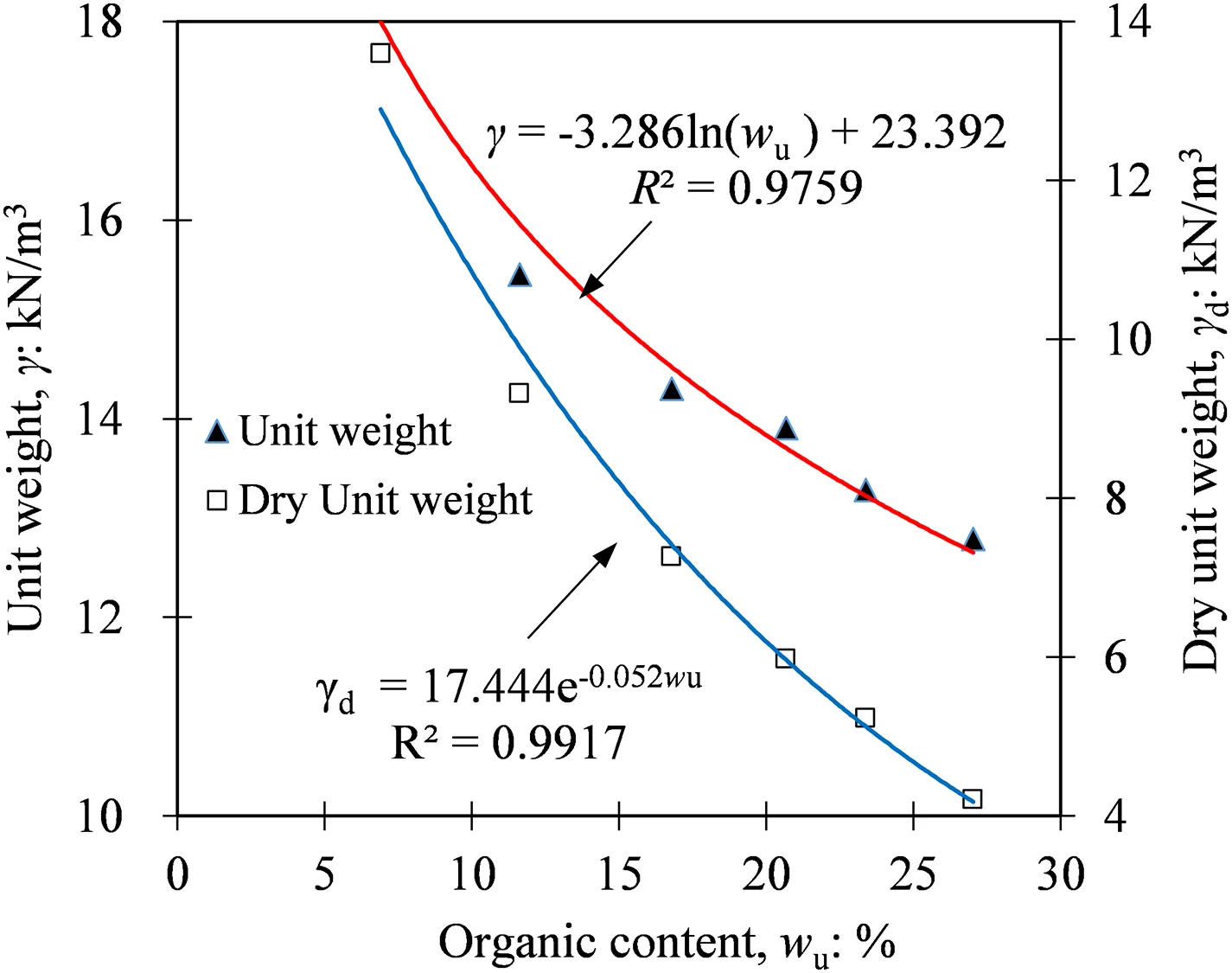
Unit weight (*γ*) and dry unit weight (*γ*_d_) versus organic content (*w*_u_).


$$\begin{array}{c} \gamma =-\text{3}.\text{2}\text{8}\text{6}ln(w_{u})+\text{2}\text{3}.\text{3}\text{9}\text{2} \end{array}$$ (1)

Eq (1) uses the units of kN/m³ for unit weight (*γ*) and % for organic content (*w*_u_). The relevant indicators for correlation analysis are as follows (including the number of samples *n*, correlation coefficient *R*, *p*-value, standard error *SE*_r_, and 95% confidence interval *CI*): *n* = 6, *R*=−0.9978, *p* < 0.001, *SE*_r_ = 0.719, Shapiro-Wilk *p* = 0.425 > 0.05, Durbin-Watson 1.5 < *d* = 1.78 < 2.5. The 95% CI for the slope is [−3.707, −2.865]. These results confirm that unit weight decreases as a logarithmic function with increasing organic content. The fit is satisfactory, with high statistical significance and excellent predictive accuracy. Organic matter has a lower particle density than mineral solids, retains more water, and deforms elastically under load. As a result, higher organic content generally corresponds to lower dry unit weight. The high correlation coefficient shows that, within this range of high water content where the effect of water has been normalised by the liquid limit, the logarithmic-law relationship describes the data well. The data point at 5% organic content deviates from the general trend, possibly because a small amount of organic matter improved the particle size distribution, or because the sample contained denser minerals. Overall, Fig 6 supports the view that for highly organic soils, organic content is the main factor controlling dry unit weight, and that conventional compaction criteria developed for inorganic soils are not directly transferable.

Regression analysis yielded the following regression equation for dry unit weight as a function of organic content.


$$\begin{array}{c} \gamma_{d}=\text{17.444}e^{\left(-\text{0}.\text{0}\text{5}\text{2}wu\right)} \end{array}$$ (2)

In Eq (2), *γ*_*d*_ denotes the dry unit weight, expressed in kN/m³. The dry unit weight of organic soils decays exponentially with increasing organic content. The correlation analysis provided the following statistical parameters: *n* = 6, *R*=−0.9993, *p* < 0.001, *SE*_r_ = 0.610, the 95% confidence interval for the slope is [−6.629, −5.897], Shapiro-Wilk *p* = 0.665 > 0.05, Durbin-Watson 1.5 < *d* = 1.69 < 2.5. The 95% confidence interval for the intercept is [24.317, 26.243]. Residual analysis indicates that the residuals are normally distributed and independent of one another. The exponential model satisfies all statistical assumptions, and the regression relationship exhibits high reliability.

The relationship between organic content and the two Atterberg limits (liquid limit and plastic limit) of sludge is illustrated in Fig 7. A clear trend can be observed: both limits rise with increasing organic content. The regression analysis produced the following formulas correlating liquid limit and plastic limit with organic content, respectively.

**Fig 7:**
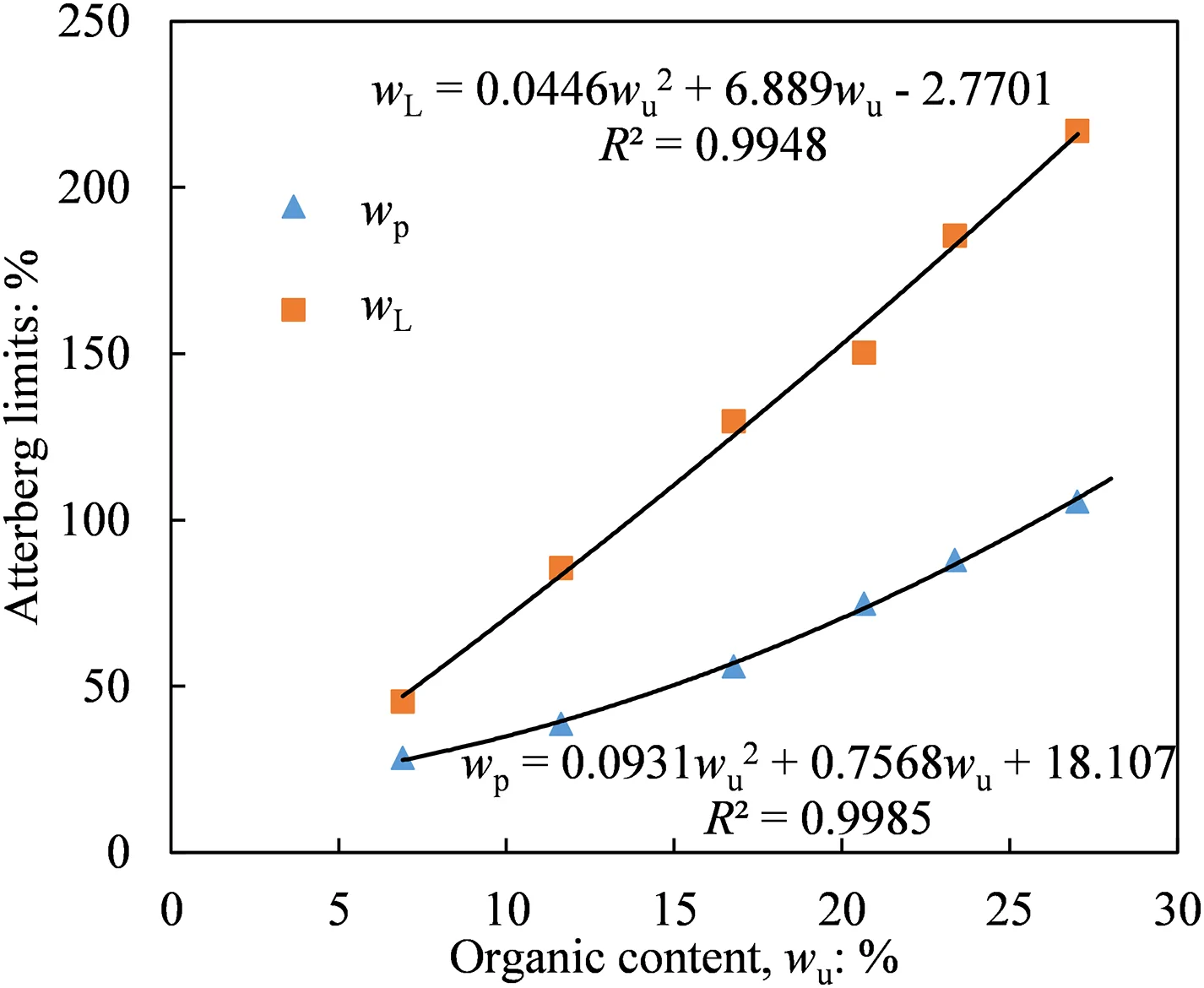
Liquid limit (*w*_L_) and plastic limit (*w*_p_) versus organic content (*w*_u_).


$$\begin{array}{c} w_{L}=\text{0}.\text{0}\text{44}\text{6}{w_{u}}^{\text{2}}+\text{6}.\text{8}\text{8}\text{9}w_{u}-\text{2}.\text{770}\text{1} \end{array}$$ (3)

$$\begin{array}{c} w_{p}=\text{0}.\text{0}\text{9}\text{31}{w_{u}}^{\text{2}}+\text{0}.\text{75}\text{6}\text{8}w_{u}+\text{1}\text{8}.\text{1}\text{0}\text{7} \end{array}$$ (4)

In Eqs (3) and (4), *w*_L_ and *w*_p_ represent the liquid limit and plastic limit, respectively, both expressed in %. For Eq (3), the indicators of correlation analysis are as follows: *n* = 6, *R* = 0.9997, *p* < 0.0001, *SE*_r_ = 1.881, Shapiro-Wilk *p* = 0.809 > 0.05, Durbin-Watson 1.5 < *d* = 2.49 < 2.5. The 95% confidence intervals: quadratic term coefficient [0.265, 0.397], linear term coefficient [−2.058, 3.246] and intercept [−24.548, 27.596]. The quadratic coefficient was statistically significant (p = 0.0001, 95% CI [0.265, 0.397]), confirming the curvature. The quadratic polynomial model satisfies all statistical assumptions, and the regression relationship is highly reliable. For Eq (4), the indicators of correlation analysis are as follows: *n* = 6, *R* = 0.9998, *p* < 0.0001, *SE*_r_ = 1.176, Shapiro-Wilk *p* = 0.810 > 0.05, Durbin-Watson 1.5 < *d* = 2.19 < 2.5. The 95% confidence intervals for the regression coefficients are as follows: quadratic term coefficient [0.160, 0.196], linear term coefficient [−1.593, 0.001], and intercept [10.716, 26.450]. The quadratic coefficient was statistically significant (p = 0.0001, 95% CI [0.160, 0.196]), confirming the curvature. The quadratic polynomial model satisfies all statistical assumptions, and the regression relationship is highly reliable. Fig 8 shows the distribution of soil samples #1 to #6 on the Casagrande plasticity chart. As can be observed, all six specimens are classified as organic silt with high liquid limit, lying below the A-line and to the right of the B-line.

**Fig 8:**
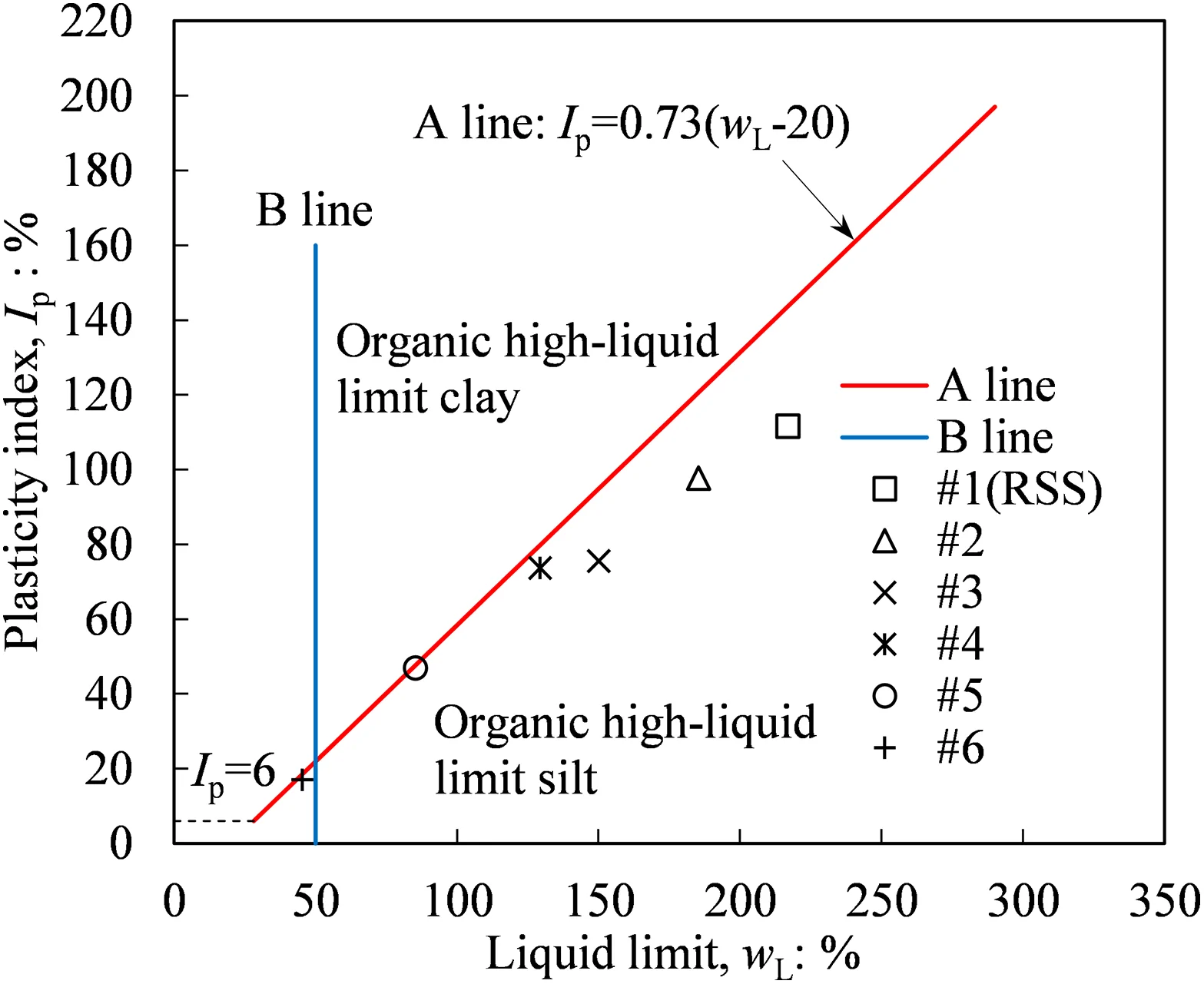
Distribution of sludge specimens on the Casagrande plasticity chart.

### Influence of water content on sludge strength

Specimens A, B, C, and D were subjected to UCS tests, and their stress-strain relationship curves are displayed in Figs 9–12. For organic soil specimen A, it can be seen from Fig 9 that samples with a normalized water content *w*_0_/*w*_L_ < 0.47 exhibit strain-softening behavior in their stress–strain response, whereas those with *w*_0_/*w*_L_ > 0.57 show strain-hardening behavior. For organic soil specimen B, Fig 10 shows that only the sample with *w*_0_/*w*_L_ = 0.39 displays strain-softening behavior, while all other samples with higher normalized water contents exhibit strain-hardening behavior. For organic soil specimen C, it can be seen from Fig 11 that samples with a normalized water content *w*_0_/*w*_L_ < 0.44 exhibit strain-softening behavior in their stress–strain response, whereas those with *w*_0_/*w*_L_ > 0.53 show strain-hardening behavior. For organic soil specimen D, it can be observed from Fig 12 that samples with a normalized water content *w*_0_/*w*_L_ < 0.50 display strain-softening behavior, while those with *w*_0_/*w*_L_ > 0.64 exhibit strain-hardening behavior. In general, the stress‑strain behaviour of sludge transitions from strain softening to strain hardening at *w*_0_/*w*_L_ = 0.45 − 0.55. The mechanism underlying this transition is discussed below: at lower normalized water contents, the soil matrix is densely packed with a well‑developed flocculated fabric; upon shearing, the progressive breakdown of inter‑particle bonds and reorientation of particles along the failure plane lead to a loss of post‑peak strength, that is strain‑softening. As *w*_0_/*w*_L_ increases beyond 0.5, the initial fabric becomes more dispersed and the inter‑particle contacts are more lubricated, so that particle rearrangement and pore‑space compression can occur without a distinct peak, resulting in a strain‑hardening response.

**Fig 9:**
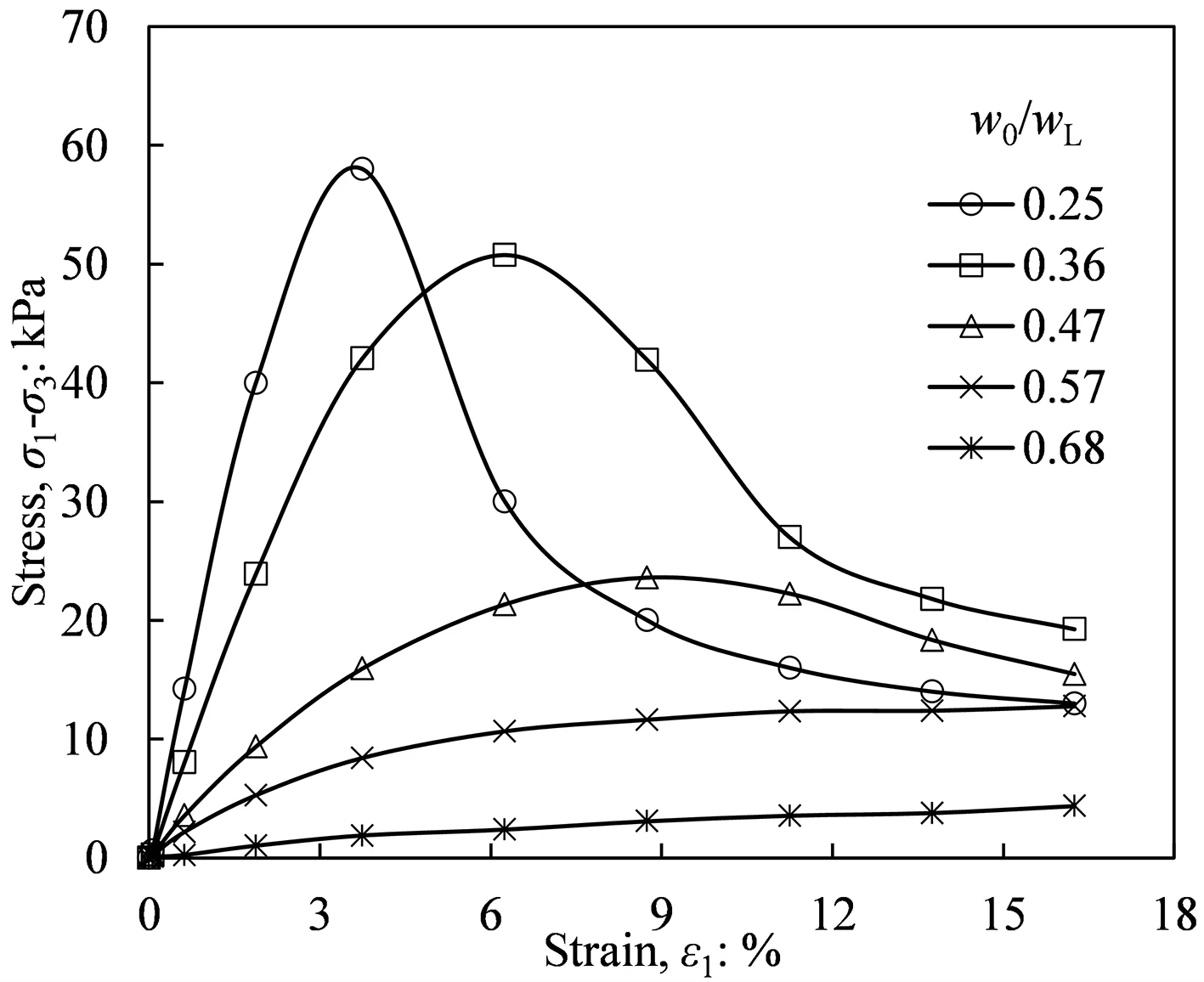
Stress-strain curves for Specimen A at varying normalized water contents.

**Fig 10:**
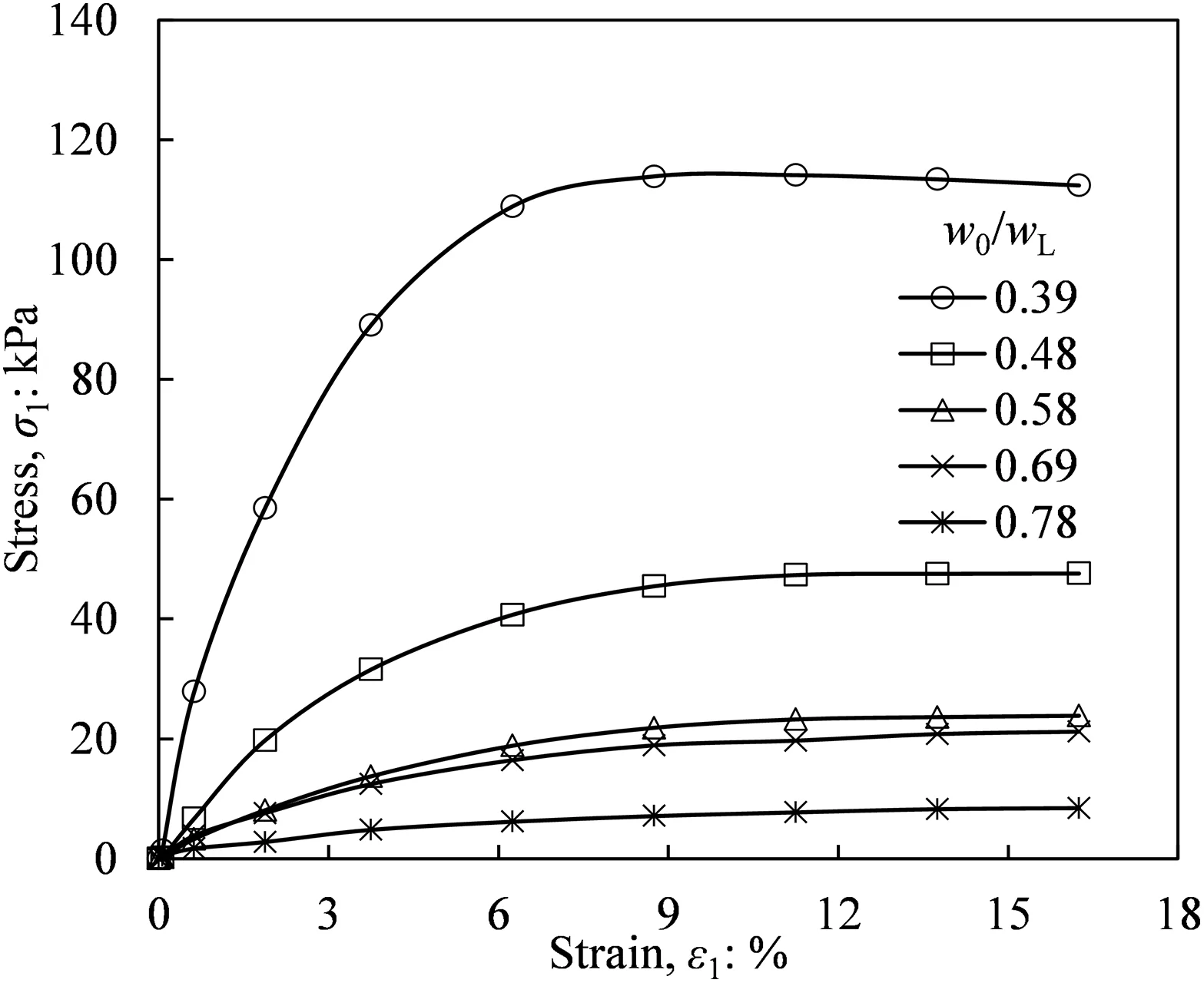
Stress-strain curves for Specimen B at varying normalized water contents.

**Fig 11:**
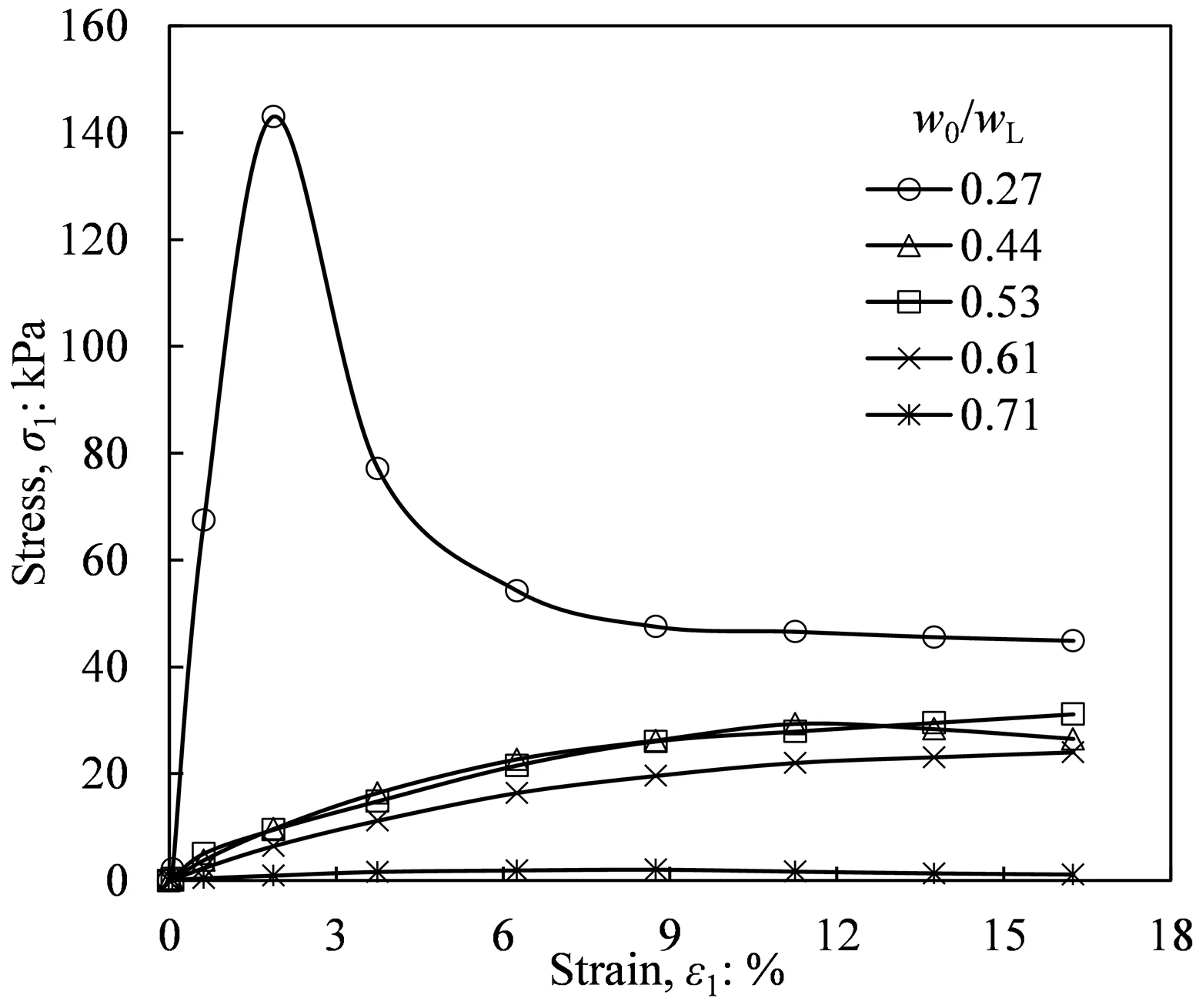
Stress-strain curves for Specimen C at varying normalized water contents.

**Fig 12:**
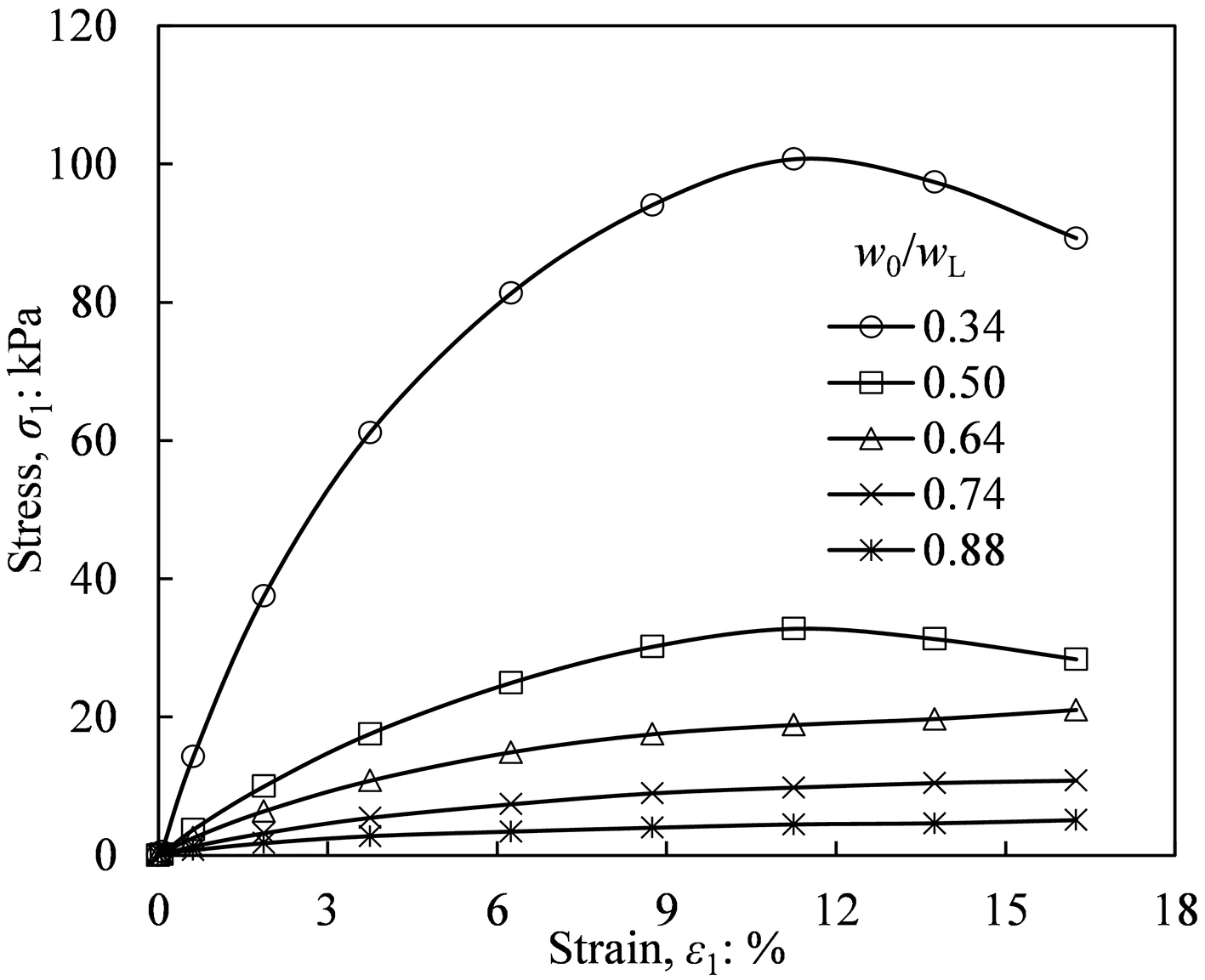
Stress-strain curves for Specimen D at varying normalized water contents.

Based on a comprehensive analysis of the stress−strain curves of the four organic soil samples A, B, C and D in Figs 9−12, it can be inferred that the critical normalized water content *w*_0_/*w*_L_ for the transition from strain-softening to strain-hardening behavior of the four organic soils ranges from 0.45 to 0.55. To investigate the variation of UCS of organic soils with normalized water content, regression analysis was performed by integrating the UCS test data of four organic high-liquid-limit silts (A, B, C and D) with published data on organic high-liquid-limit clays reported by O’Kelly (2005) [36]. As shown in Fig 13, a strongly fitted relationship was obtained between UCS and *w*_0_/*w*_L_ for the organic soils. The correlation equation is as follows.

**Fig 13:**
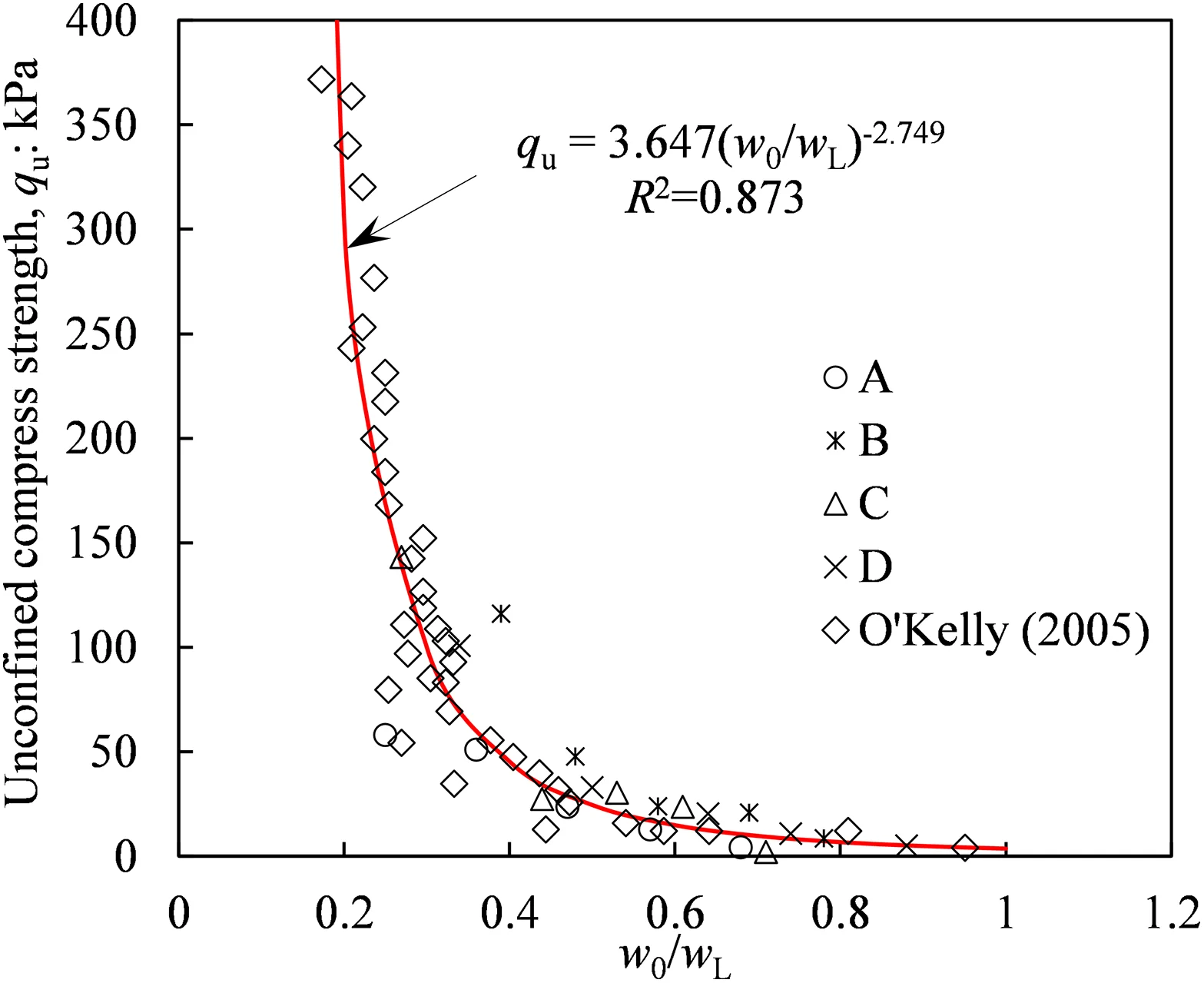
UCS (*q*_u_) versus normalized water content (*w*_0_/*w*_L_) for the sludge.


$$\begin{array}{c} q_{u}=\text{3}.\text{6}\text{4}\text{7}(w_{\text{0}}/w_{L}{)}^{-\text{2}.\text{7}\text{4}\text{9}} \end{array}$$ (5)

In Eq (5), *q*_u_ denotes the UCS, expressed in kPa. The indicators of correlation analysis are as follows: *n* = 58, *R*=−0.935, *p* < 0.001, *SE*_r_ = 0.050, 95% *CI* [−0.962, −0.892]. These results indicate an excellent fitting performance and high statistical significance, meaning the power-law regression model yields strong predictive accuracy. This also confirms that describing the variation in UCS with *w*_0_/*w*_L_ using a power function delivers reliable prediction accuracy and effectiveness. Given that the absolute value of the power exponent in Eq (5) exceeds 2, strength is highly sensitive to changes in water content. Even a small increase in water content leads to a sharp reduction in strength, particularly at low water contents. Eq (5) can be used to predict the UCS of municipal sewage sludge with a moisture content (*w*_0_/*w*_L_) between 0.2 and 1.0 and an organic content between 17% and 50%.

Fig 13 incorporates experimental data of high‑plasticity organic clay from O’Kelly (2005). The material reported by O’Kelly (2005) was anaerobically digested sewage sludge, with an organic content *w*_u_ = 55%, liquid limit *w*_L_ = 315%, and plastic limit *w*_p_ = 55%. By comparison, the organic soil tested in the present work is high‑plasticity silt, which is also sourced from anaerobically digested sewage sludge, with organic content *w*_u_ ranging from 17.08 − 45.50%, liquid limit w_L_ from 136.3 − 249.00%, and plastic limit *w*_p_ from 55.5 − 101.29%. The test procedures for organic content, liquid limit, plastic limit and water content in our study followed the methods reported by O’Kelly (2005) [36]. Given the substantial similarities in sludge origin and testing protocols between the two datasets, we included O’Kelly (2005) data in the fitting to broaden the applicability of the proposed model.

The unconfined compressive strength of organic soils exhibits a monotonic decrease with increasing normalized water content *w*_0_/*w*_L_ as shown in Fig. 13. Unlike inorganic clays, where a bell-shaped relationship with a distinct peak is typically observed near *w*_0_/*w*_L_ from 0.4 to 0.6, the organic soils in this study show no such peak. At *w*_0_/*w*_L_ = 0.15, the strength is approximately 375 kPa, falling to below 10 kPa as *w*_0_/*w*_L_ approaches 0.90. This difference arises because the strength of organic soils is governed primarily by matric suction and the entanglement of fibrous organic matter, rather than by frictional interlocking between mineral particles. As water content increases, matric suction diminishes rapidly, and the high porosity and elastic nature of organic matter prevent the formation of a stable particle skeleton. The data also compare closely with those reported by O’Kelly (2005), confirming that the observed trend is consistent across different organic soil types. These findings imply that conventional strength criteria developed for inorganic soils are not directly applicable to highly organic soils, and that water content control is more critical than attempting to improve strength through compaction or stabilisation alone.

### Influence of organic matter on sludge strength

Figs 14–17 present the stress–strain curves of six organic soil specimens #1 to #6 prepared by incorporating DSP into RSS under confining pressures of 0, 100, 200, and 300 kPa, respectively. As shown in Figs 14–17, all six specimens display a strain-hardening behavior. Stress–strain curves for soils with low organic content lie above those with high organic content, indicating higher strength in low-organic soils. Notably, curves for specimens #3 and #4 diverge noticeably with a distinct “jump,” which suggests that strength dropped sharply as organic content decreased from 20.67% to 16.78%. This abrupt reduction, however, became less significant with increasing confining pressure.

**Fig 14:**
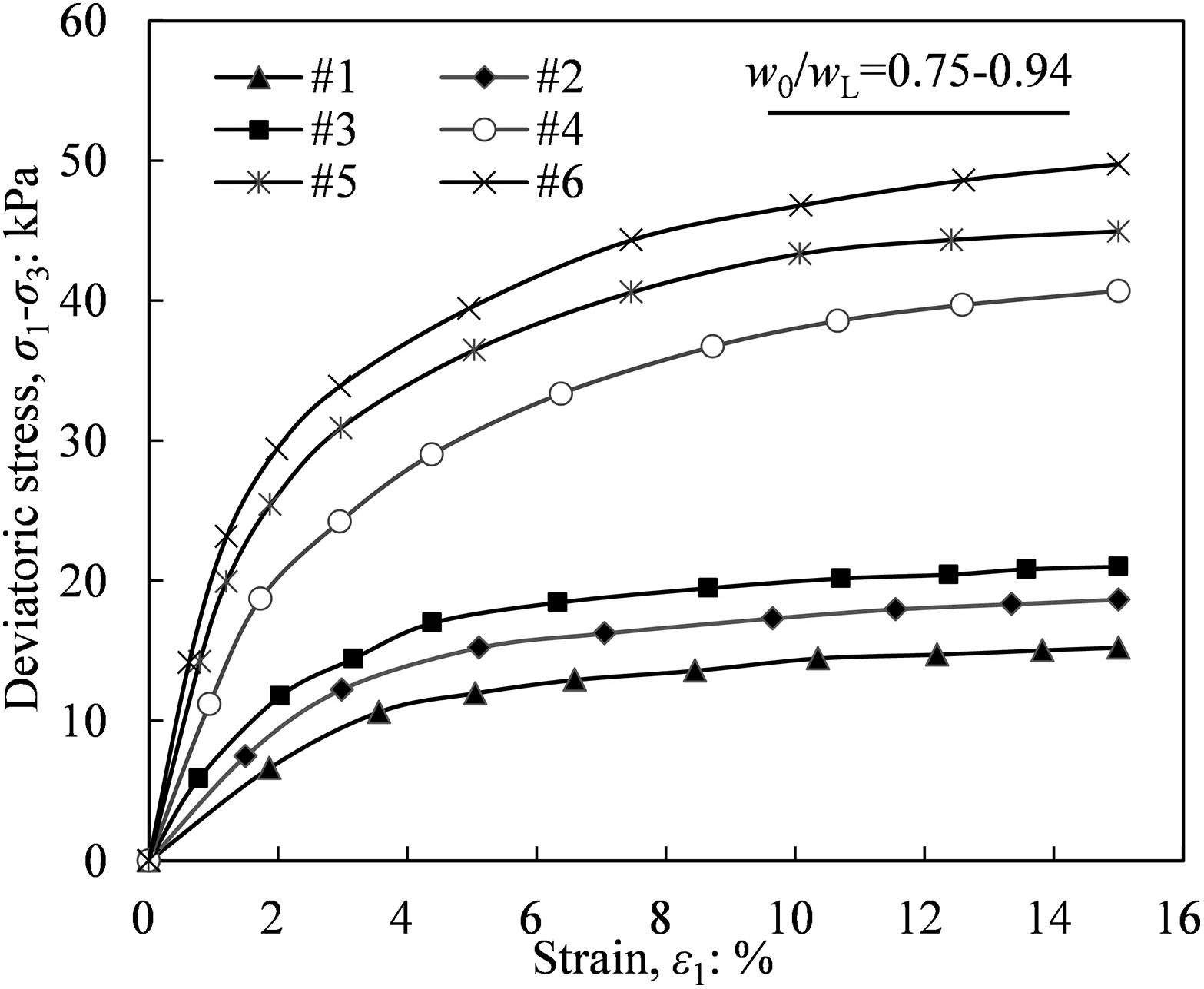
Strain−strain curves of sludge specimens under unconfined condition (σ_3_ = 0 kPa).

**Fig 15:**
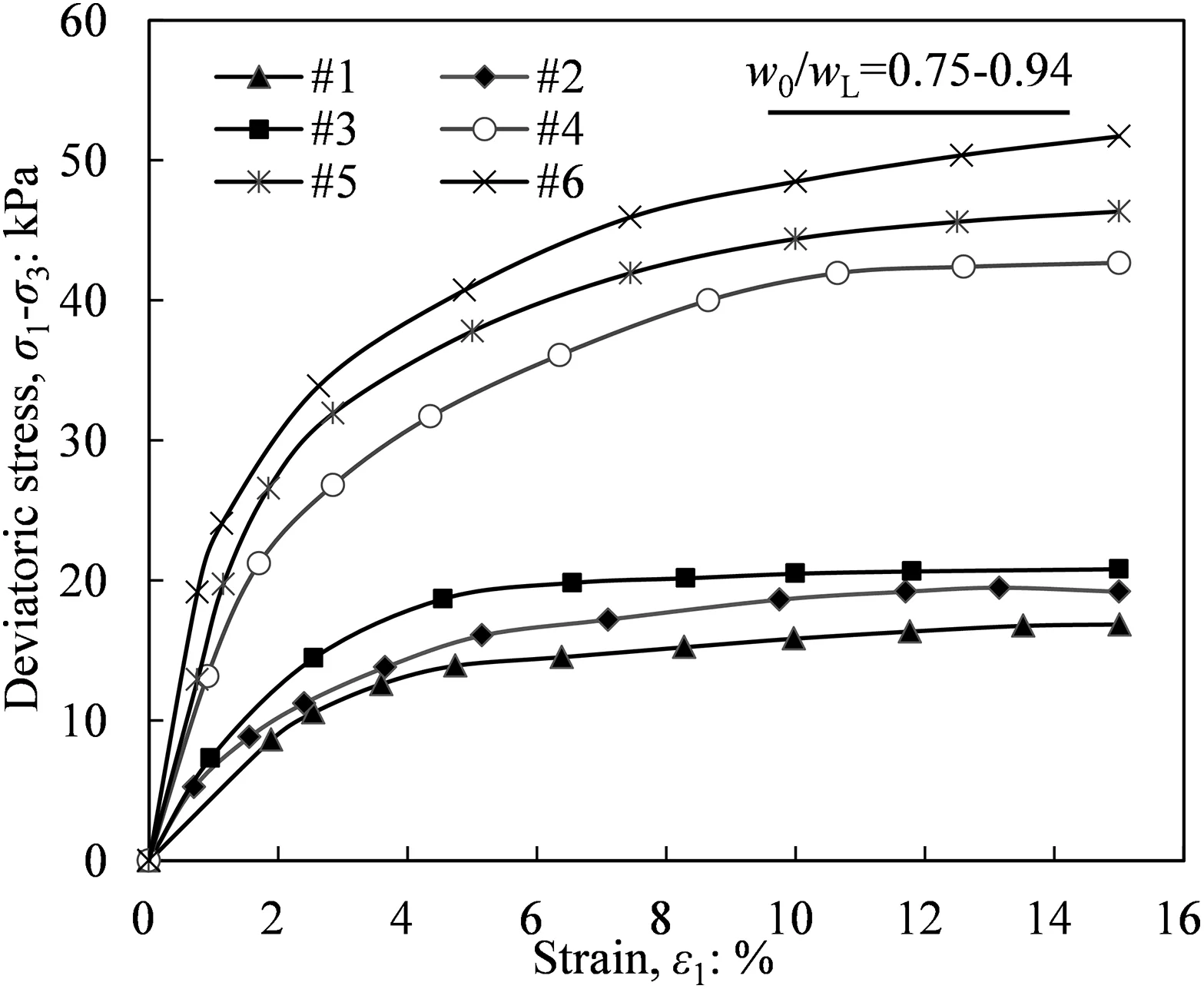
Stress−strain curves of sludge specimens under confining pressure σ_3_ = 100 kPa.

**Fig 16:**
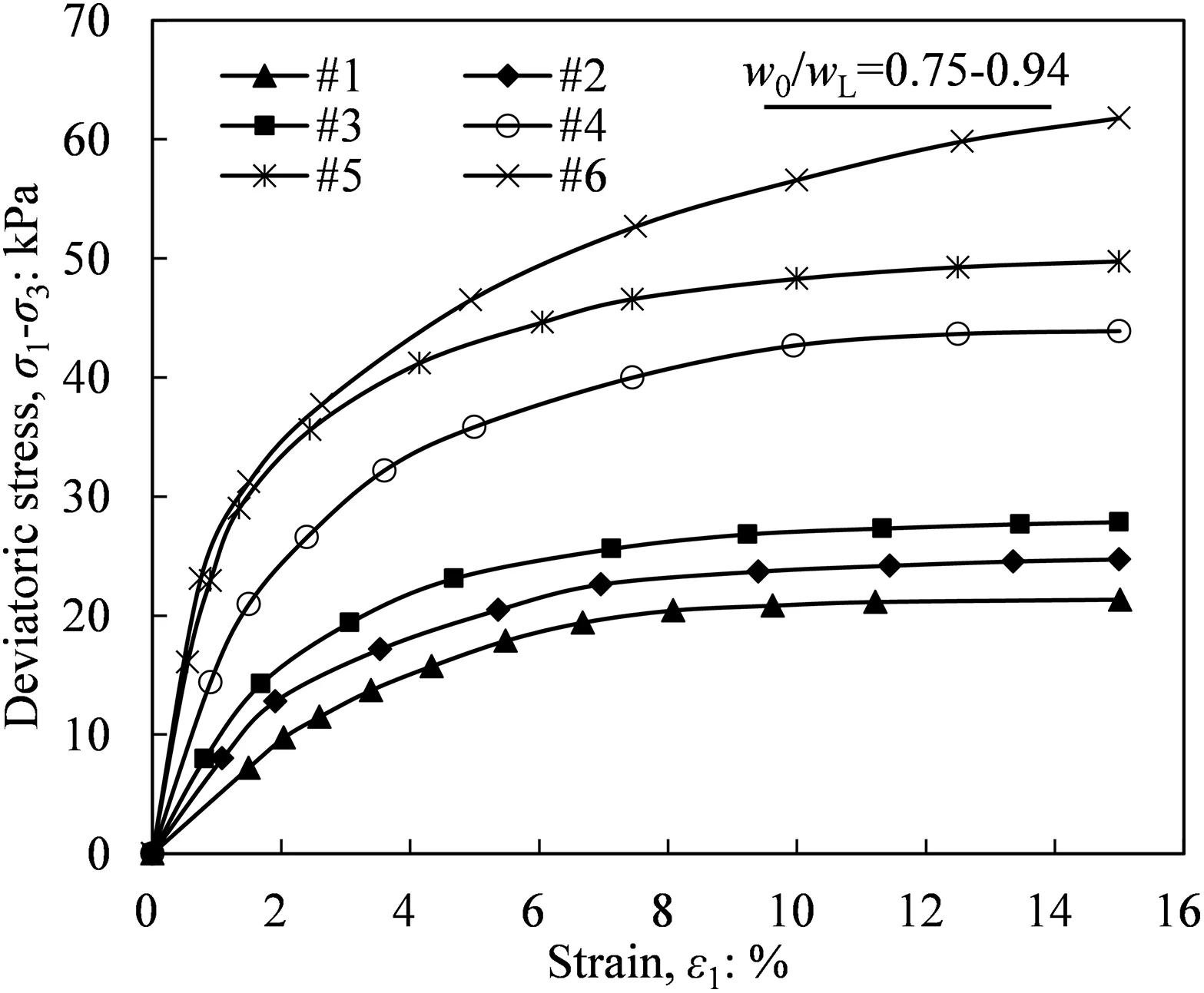
Stress−strain curves of sludge specimens under confining pressure σ_3_ = 200 kPa.

**Fig 17:**
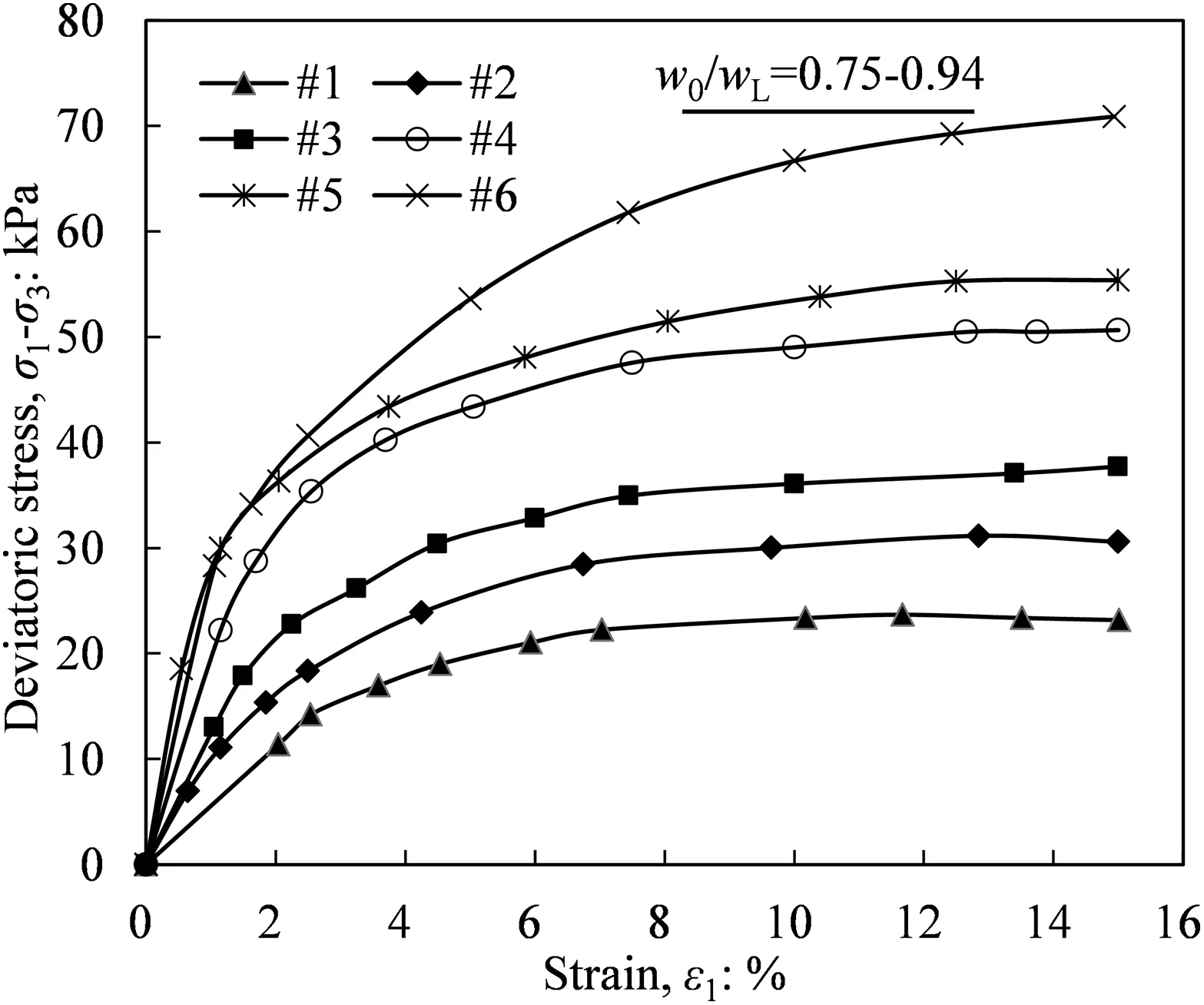
Stress-strain curves of sludge specimens under confining pressure σ_3_ = 300 kPa.

Hardening stress–strain curves do not exhibit a distinct peak stress. For such cases, the stress at 15% strain is defined as the failure stress. Based on the stress–strain behavior shown in Fig 14, the relationship between UCS and *w*_u_ was derived and is plotted in Fig 18, which indicates that UCS declines linearly with increasing *w*_u_. The regression equation describing UCS as a function of *w*_u_ for organic high–liquid–limit silt, with a normalized moisture content *w*_0_/*w*_L_ ranging from 0.75 to 0.94, is given below.

**Fig 18:**
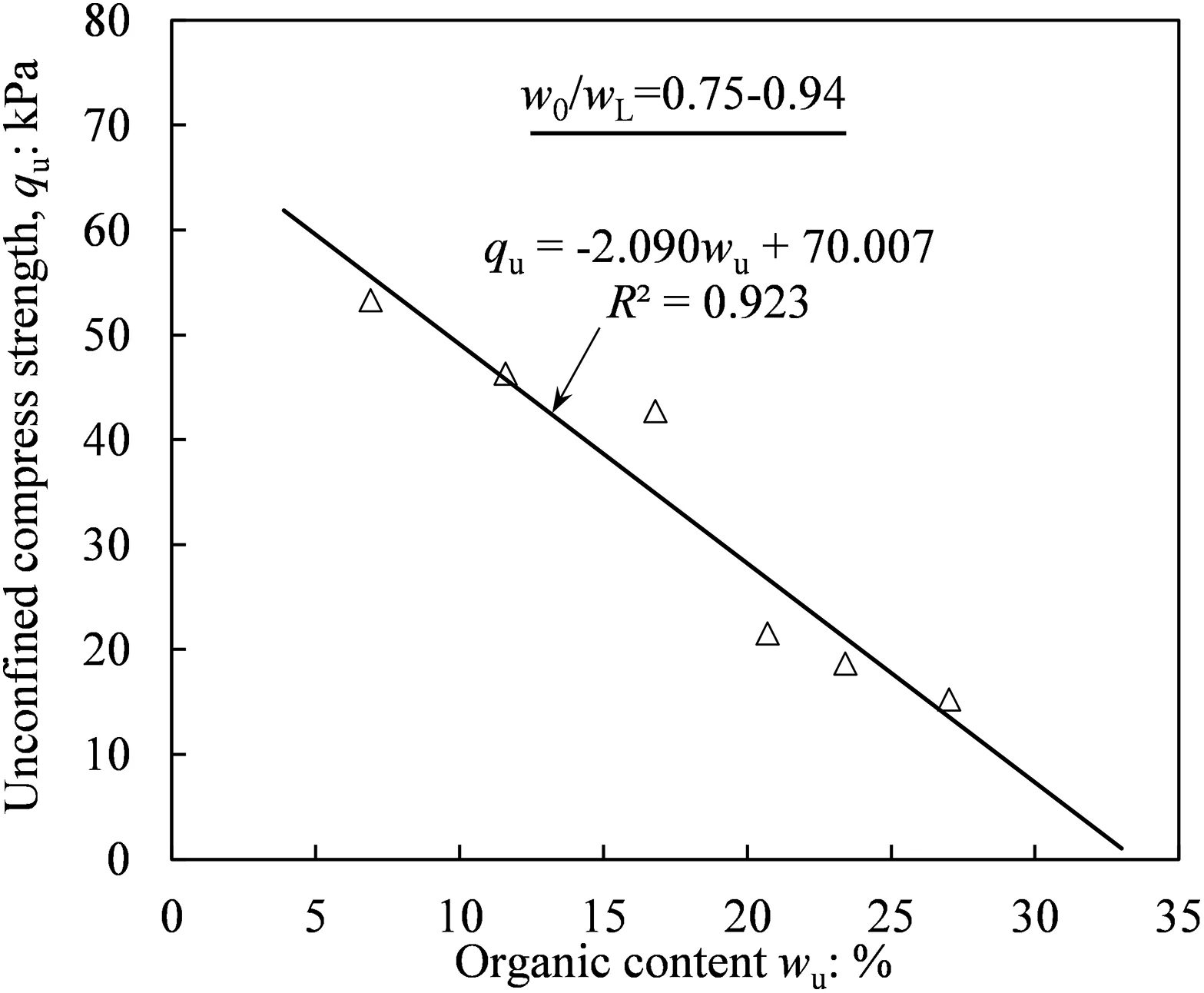
Unconfined compressive strength versus organic content.


$$\begin{array}{c} q_{u}=-\text{2}.\text{0}\text{9}\text{0}w_{u}+\text{7}\text{0}.\text{0}\text{0}\text{7} \end{array}$$ (6)

In Eq (6), *q*_u_ represents the UCS, expressed in kPa; *w*_u_ represents the organic content, expressed as a percentage. For Eq (6), the relevant indicators of correlation analysis are as follows: *n* = 6, *R*=−0.961, *p* = 0.002, *SE*_r_ = 0.139, 95% *CI* [−0.995, −0.717], Shapiro–Wilk *p* = 0.793 > 0.05, Durbin–Watson *d* = 0.92 < 1.5. Residual diagnostics indicated that the Shapiro–Wilk normality assumption was satisfied (*p* = 0.739 > 0.05). The Durbin–Watson statistic was *d* = 0.92, slightly below the lower bound of 1.5, suggesting potential serial correlation—likely attributable to the small sample size and abrupt data changes within the range *w*_u_ = 16.8–20.7. Overall, *q*_u_ exhibited a significant negative correlation with *w*_u_. Given the practical convenience for subsequent applications, the linear distribution model was retained, although its accuracy and applicability warrant further validation. The regression demonstrates a statistically significant negative association between organic content and UCS, with an excellent goodness of fit. Eq (6) can be used to predict the unconfined compressive strength of municipal sewage sludge with the organic high-liquid-limit type and an organic content ranging from 5% to 30%.

Using Figs 15−17, and similarly taking the deviatoric stress (*σ*_1_ − *σ*_3_)_f_ corresponding to a strain of 15% as the failure stress, we obtained the relationships between failure stress and organic content under confining pressures of 100, 200, and 300 kPa, as demonstrated in Fig 19. As shown in Fig 19, the failure stress (*σ*_1_-*σ*_3_)_f_ of the organic soil decreases with increasing organic content under three confining pressures. Evidently, within the organic content range of 17–21%, the failure stress under the three confining pressures decreases rapidly with increasing organic content, implying that this organic‑content interval may represent a transition zone for structural degradation of organic soils.

**Fig 19:**
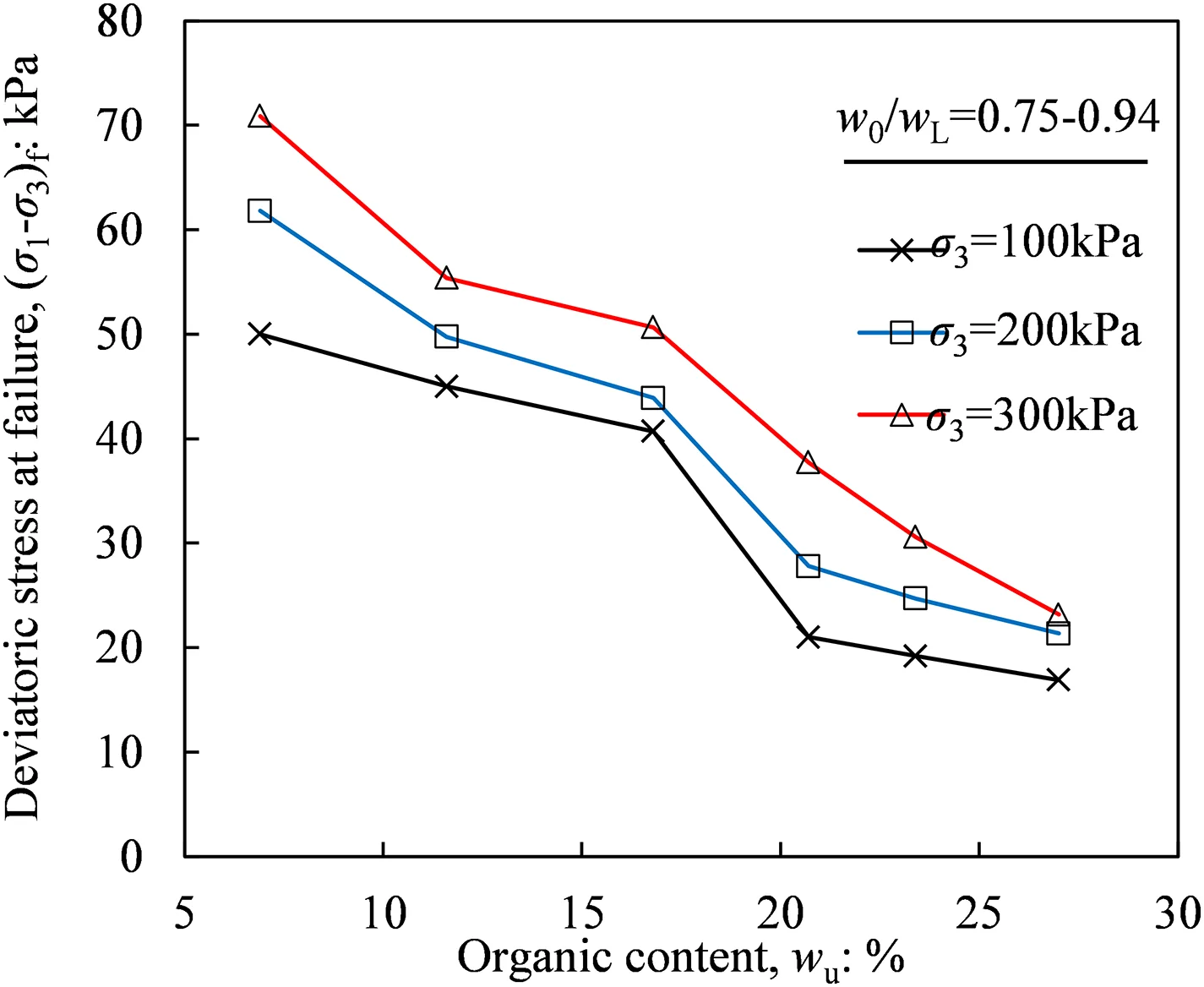
Failure stress versus organic content at confining pressures of 100, 200 and 300 kPa.

Fig 20 illustrates how organic content affects the shear strength parameters—namely cohesion (*c*_u_) and angle of internal friction (*φ*_u_)—both of which tend to decrease as organic content increases. The *φ*_u_ value from unconsolidated undrained triaxial tests is very low, ranging from 1° to 3°. The *c*_u_ value declines at a comparatively slow rate from 19 kPa to 17.2 kPa when organic content is lower than 17%. When the organic content is between 17% and 21%, the *c*_u_ value decreases from 17.2 kPa to 6.15 kPa, with a sharp and significant decline. When the organic content exceeds 21%, the *c*_u_ value exhibits minimal variation as the organic content increases. In practice, soils with organic content over 21% undergo substantial plastic deformation under loading and can be considered failed per conventional failure criteria, leading to consistently low cohesion values. In other words, cohesion remains at a low level even with further increases in organic content beyond 21%.

**Fig 20:**
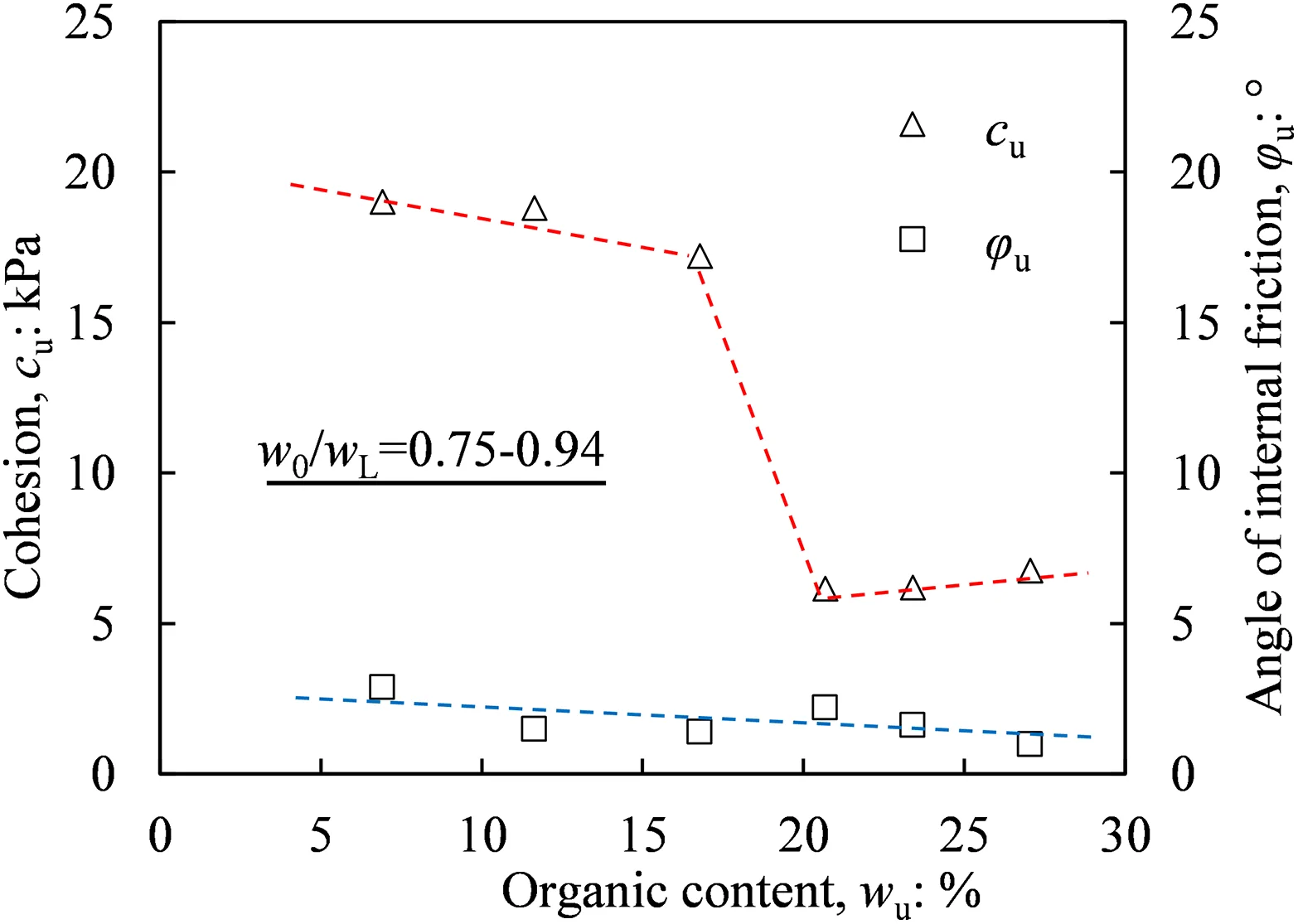
Shear strength indicator versus organic content.

As illustrated in Fig 20, the shear strength parameters of the tested organic soils are clearly correlated with organic content: the undrained cohesion *c*_u_ decreases from approximately 19.0 kPa at *w*_u_ = 5% to 7.0 kPa at *w*_u_ = 25%, whereas the undrained friction angle *φ*_u_ falls within a narrow range of 1°–3° and shows a mild declining trend with increasing organic content. Such low friction angles stem from the microstructural features of organic soils; their porous, low‑density, frequently fibrous or platy particles limit effective interparticle contact, and high in‑situ water content keeps the soil nearly saturated, whereby pore‑water pressure bears most of the applied normal stress and little effective stress is available to develop frictional resistance. Consequently, shear resistance is dominated by the viscous resistance of the organic matrix and fibre stretching‑breakage behaviour rather than true interparticle friction. The cohesion reduction at higher organic content results from progressive dilution of mineral bonds by poorly cementing organic matter; replacement of mineral fractions by organic components weakens internal bonding and produces more compressible, less structured soil. Consistent with published results for peats and organic fine‑grained soils [37,38], these observations demonstrate that low shear strength and minor friction angles are intrinsic properties of highly organic soils rather than testing artifacts.

### Mechanism analysis and discussion

Figs 21–26 present the SEM micrographs of organic soils with different organic contents. According to the SEM micrographs and the variation law of organic content versus dry unit weight in Fig 6, we inferred the mechanism causing the distribution of cohesion with organic content observed in Fig 20. At low organic contents (*w*_u_ < 17%): dry unit weight declines slowly, the soil is still dominated by a mineral particle skeleton, and organic matter acts only to fill interparticle voids. This causes only limited weakening of interparticle cementation and interlocking, so cohesion decreases gradually from 19 kPa to 17.2 kPa. Based on the SEM micrographs of organic soil shown in Figs 25-26, combined with the trend in Fig 6 that dry unit weight decreases with increasing organic content, the mechanism underlying the higher strength of specimens with low organic content can be inferred as follows. When the proportion of DSP is relatively high, rigid inorganic mineral particles dominate and form a stable skeleton interlocking structure. Meanwhile, such specimens possess a higher dry unit weight and lower porosity, accompanied by a more compact particle arrangement and a remarkable reduction in microcracks and structural defects. A small amount of organic matter uniformly coats and cements inorganic particles to form continuous and dense aggregates. The contact mode between particles transitions from point contact to surface contact, which effectively enhances the interparticle bonding strength and structural integrity. Quantitative image analysis of the SEM micrographs (using ImageJ) further supports this interpretation: for specimens with *w*_u_ < 17%, the average pore area fraction is only 4.2–5.6%, while the number of particle‑to‑particle contacts per unit area is approximately 2.3–2.7 times higher than that in high‑organic specimens. The mean aggregate size remains above 15 µm, and the aspect ratio of pores is close to unity, indicating a predominantly equiaxed, closed‑pore structure that contributes little to stress concentration. These metrics quantitatively confirm that the mineral skeleton remains largely intact and that organic matter merely fills the voids without disrupting the load‑bearing framework.

**Fig 21:**
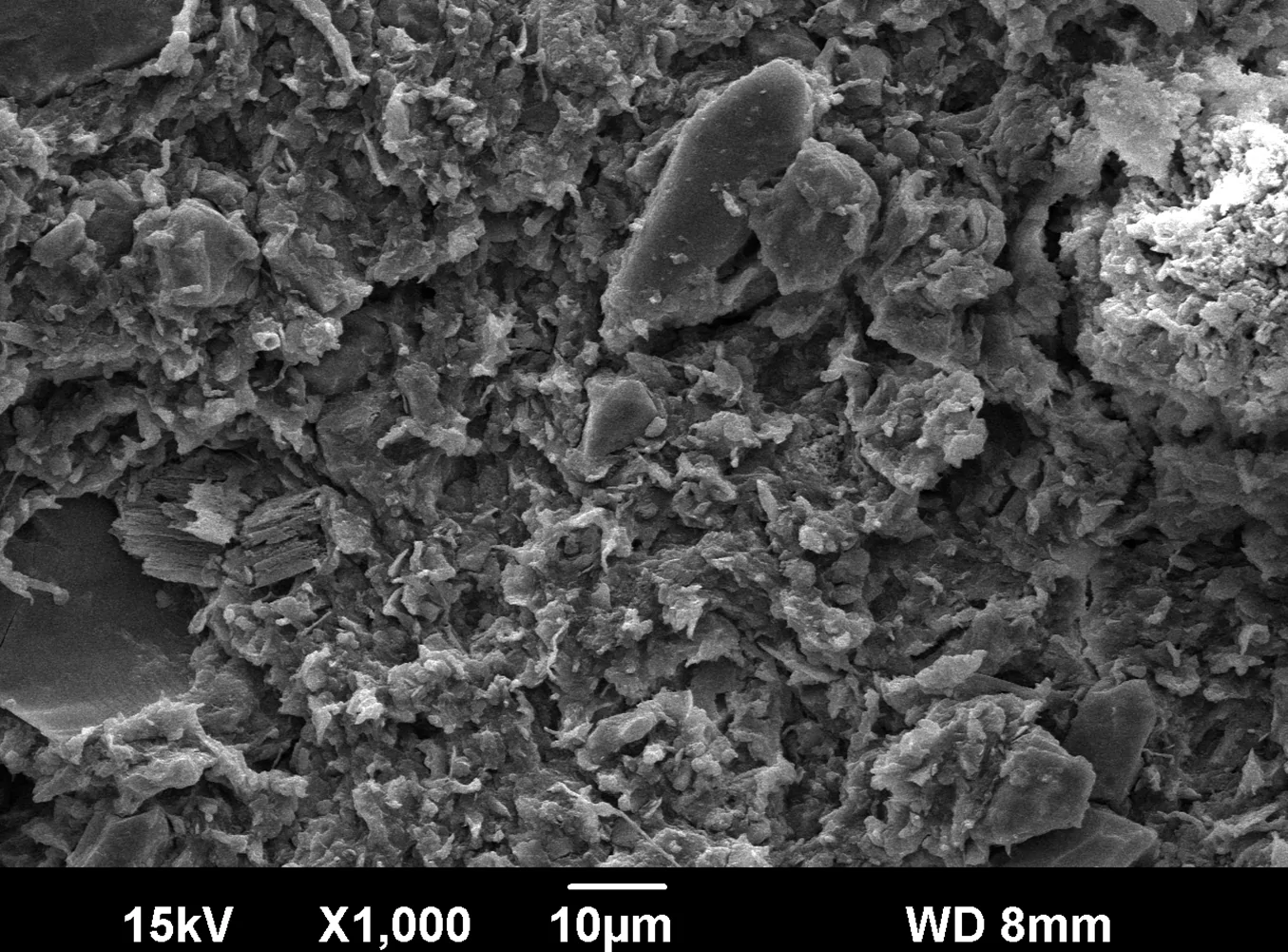
SEM image of Specimen #1 (*w*_u_ = 27.03%).

**Fig 22:**
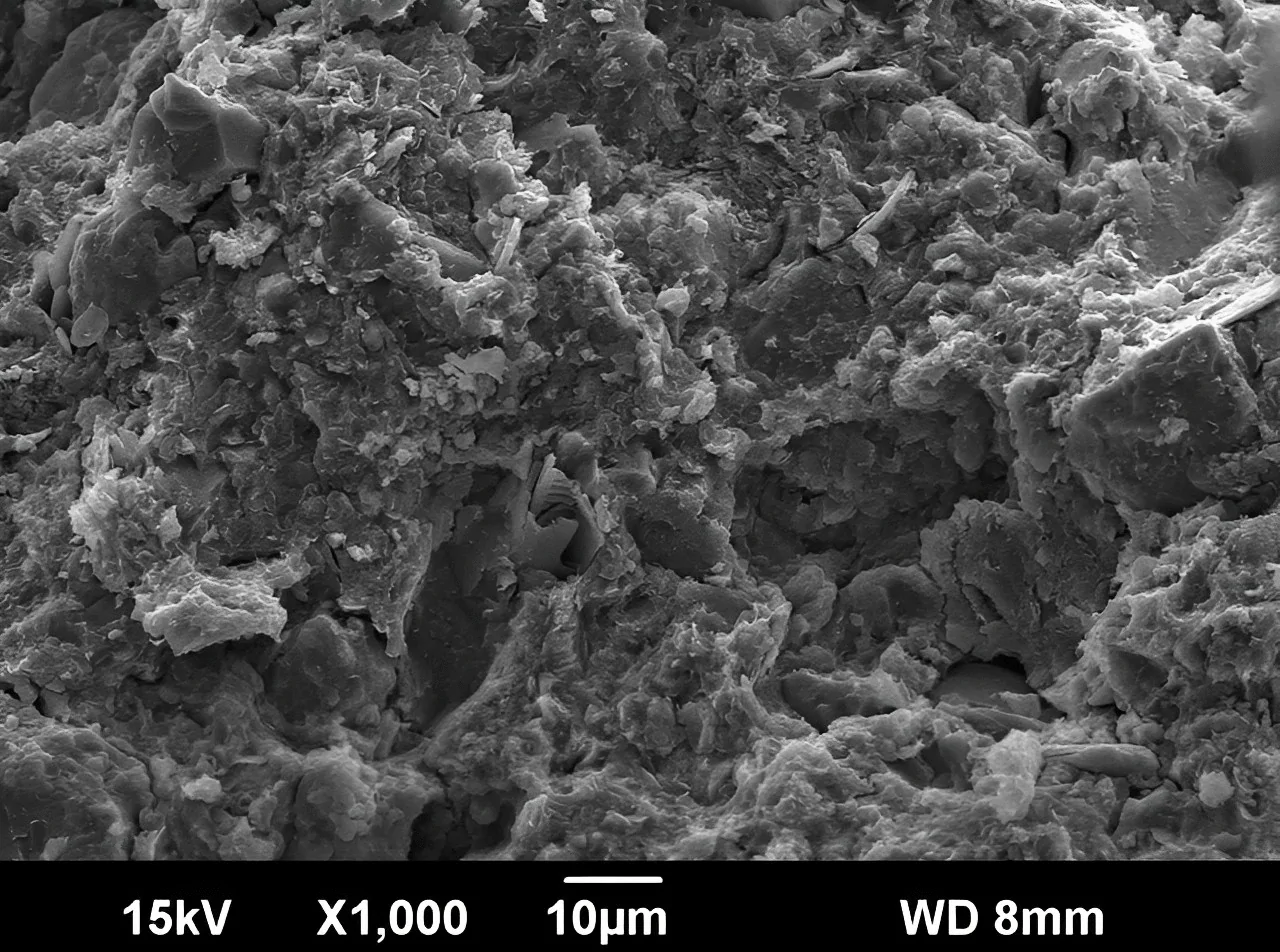
SEM image of Specimen #2 (*w*_u_ = 23.38%).

**Fig 23:**
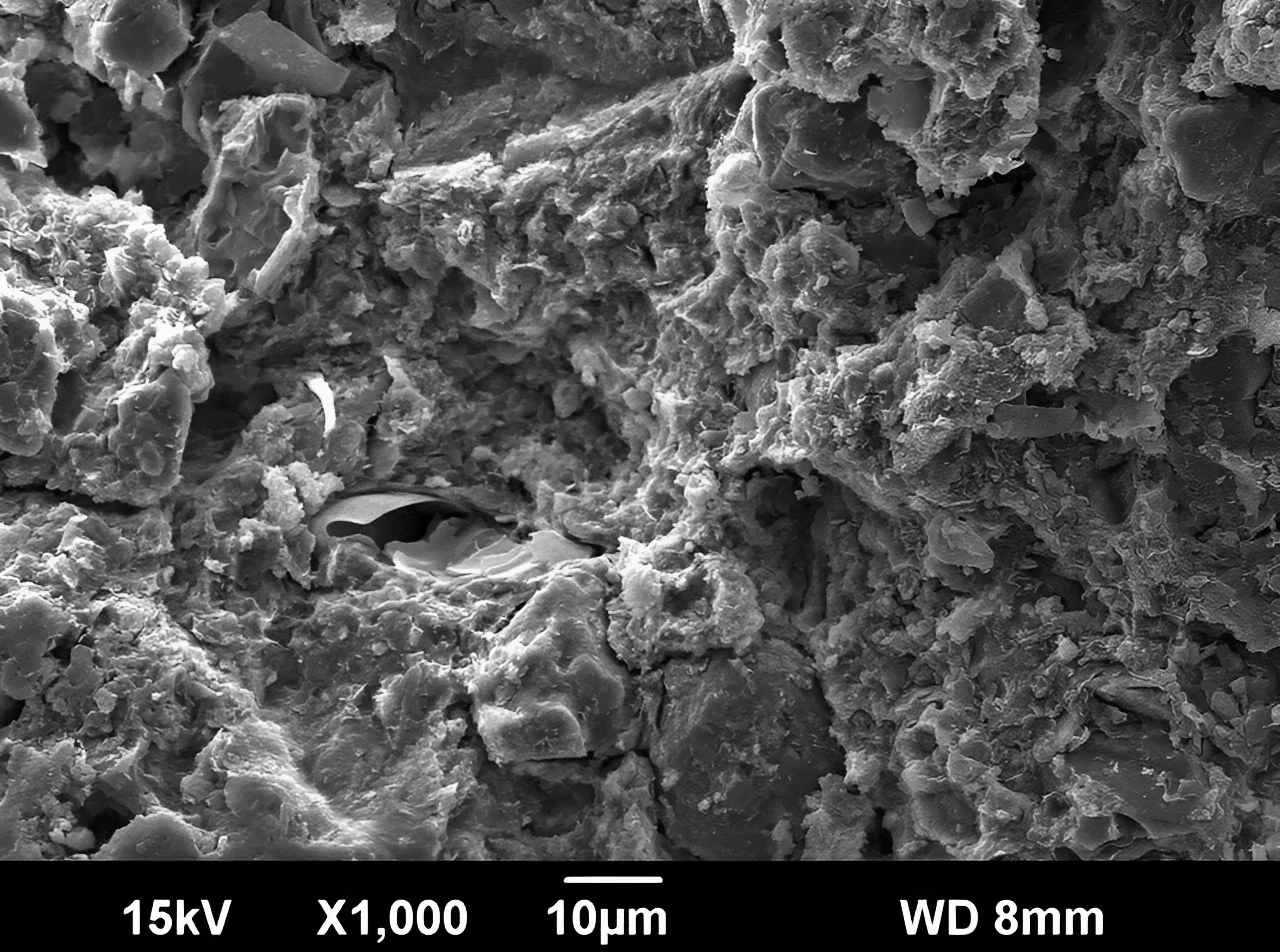
SEM image of Specimen #3 (*w*_u_ = 20.67%).

**Fig 24:**
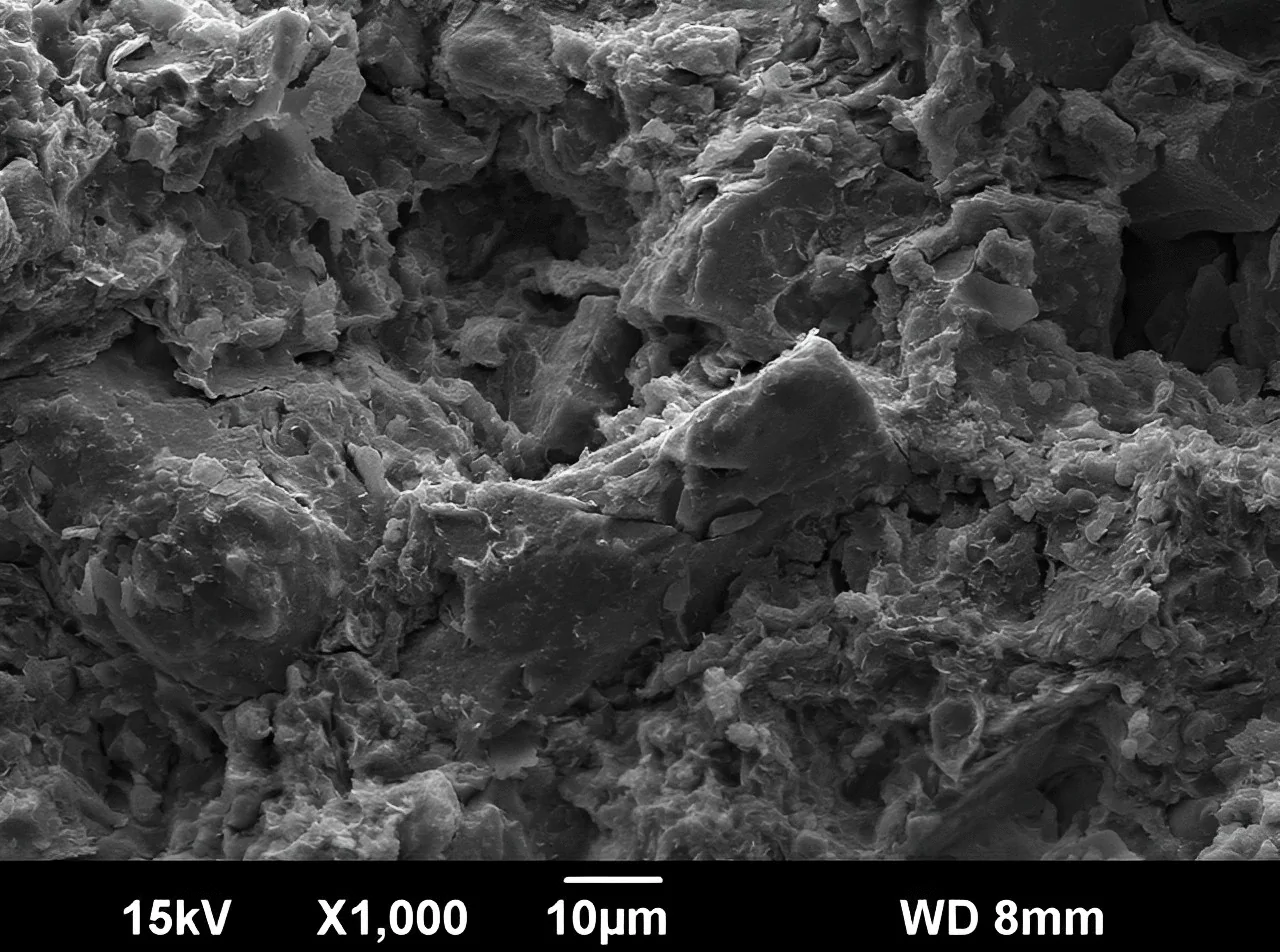
SEM image of Specimen #4 (*w*_u_ = 16.78%).

**Fig 25:**
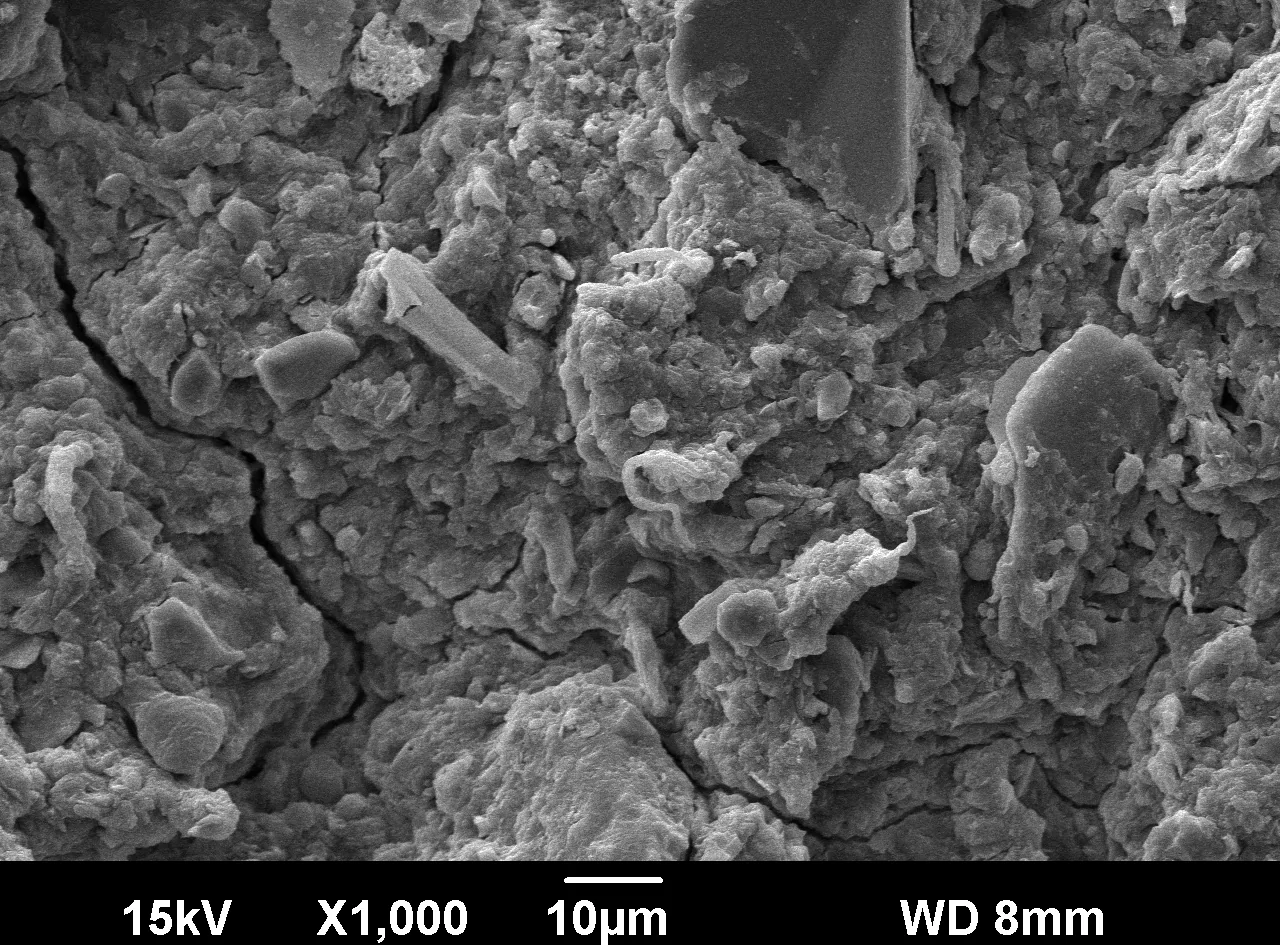
SEM image of Specimen #5 (*w*_u_ = 11.63%).

**Fig 26:**
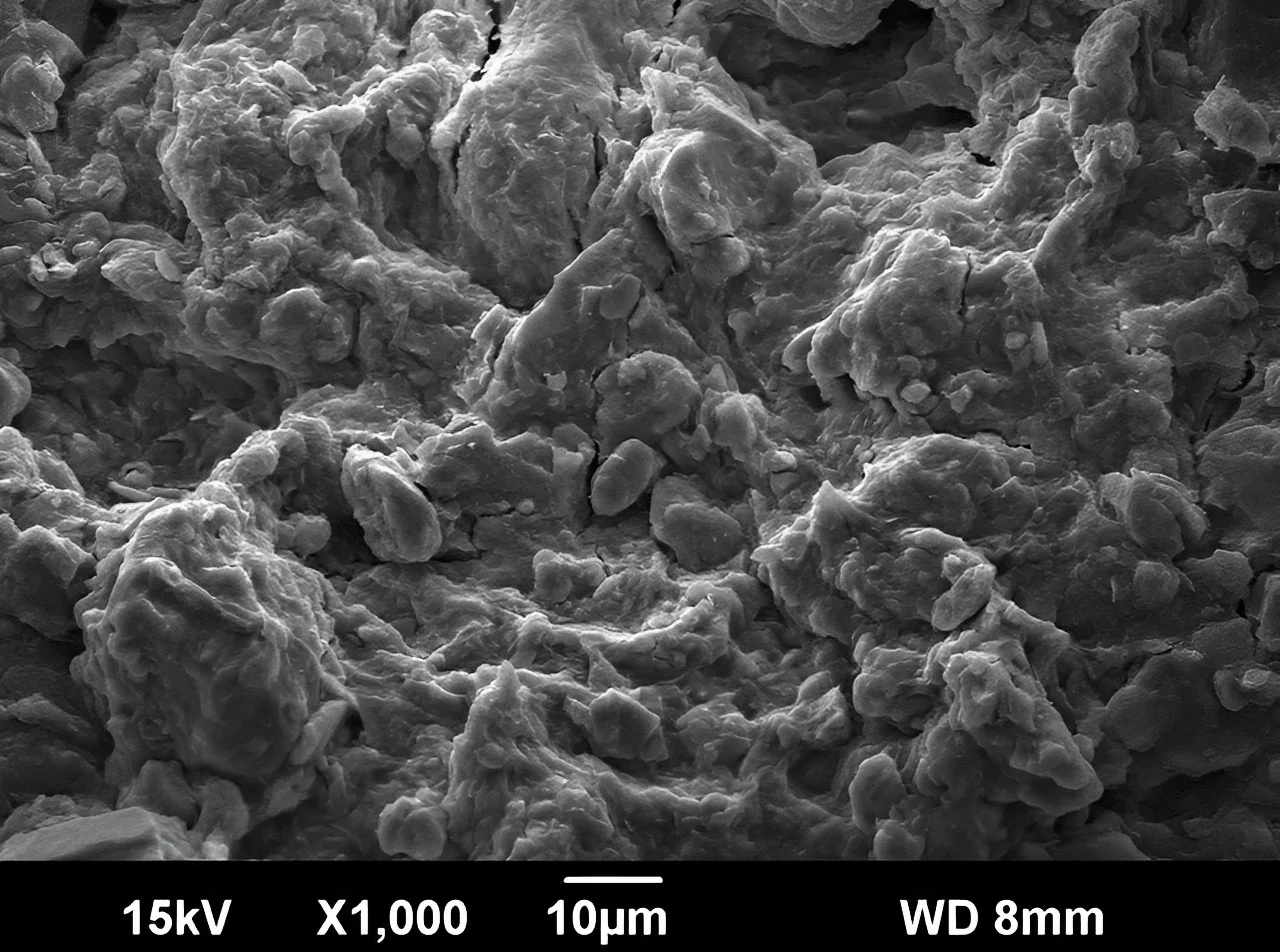
SEM image of Specimen #6 (*w*_u_ = 6.913%).

During the critical transition range (17% < *w*_u_ < 21%): as organic content increases, dry unit weight decreases rapidly. Organic matter gradually replaces mineral particles to form a continuous, weak matrix. The original particle skeleton is disrupted, the effective contact area decreases sharply, and cohesion drops precipitously to 6.15 kPa. By comparing the SEM micro-morphologies of organic soils illustrated in Figs 23−25, coupled with the trend depicted in Fig 6 wherein dry unit weight decreases with increasing organic content, the following conclusions can be drawn. As organic content increases—especially when it rises from 17% to 21%—even a slight variation in organic content not only significantly reduces the dry unit weight of organic soil and severely impairs the skeletal support function of inorganic particles but also forms a large number of soft interlayers between particles. These soft interlayers drastically diminish the frictional and interlocking effects among particles. Simultaneously, the number of internal microcracks and large pores in the specimens increases sharply, resulting in a prominent stress concentration effect, which ultimately leads to a dramatic decline in soil strength. Quantitative image analysis clearly captures this degradation: within the critical transition zone, the pore area fraction jumps from 5% to 18%, and the average pore diameter increases by nearly 3 times. Concurrently, the particle contact density drops by over 40%, and the proportion of surface‑to‑surface contacts decreases from 65% to 22%, being replaced by predominantly point‑and‑edge contacts. The fractal dimension of pore boundaries rises markedly, reflecting a more irregular and interconnected pore network. These quantitative indicators provide direct evidence for the progressive breakdown of the mineral skeleton and the emergence of a continuous organic phase that disrupts effective load transfer.

At high organic contents (*w*_u_ > 21%): dry unit weight remains at a low level, and soil structure is entirely dominated by organic matter. The high plasticity of organic matter causes significant plastic deformation under loading; according to failure criteria, the soil is close to the failure state. Cohesion stabilizes at a low level of approximately 6 kPa and no longer changes significantly with increasing organic content. The SEM micrographs from Figs 21−22 show that organic soils with high organic matter content exhibit a loose, porous microstructure. Flocculent and fibrous organic matter from sludge separates mineral particles, leading to predominantly point-to-point contacts and a poorly interlocked skeleton. The organic phase provides only weak cementation and cannot form a continuous rigid bonding network. Combined with the low dry unit weight observed in Fig 6, this porous structure results in insufficient skeleton strength, while the hydrophilic nature of organic matter further weakens inter-particle contact upon water absorption. These microstructural features jointly cause the shear strength of organic soil to stay at a relatively low level with the continuous rise of organic content. Quantitative analysis of the high‑organic specimens (Figs 21−22) reveals a pore area fraction exceeding 22–25%, with mean pore sizes larger than 8 µm and a high proportion of elongated, interconnected macropores (aspect ratio > 1.8). The particle contact density falls to less than one‑third of that observed in low‑organic samples, and over 70% of contacts are point‑to‑point, indicating a nearly absent interlocking network. The organic matrix appears as a continuous phase with low stiffness, which is corroborated by the measured increase in pore circularity—suggesting that pores are largely moulded by the soft, deformable organic matter. These quantitative features unequivocally demonstrate that the mechanical response is no longer governed by a mineral skeleton but rather by the compliant organic framework, which controls strength through its cementing action and plastic deformability rather than through frictional resistance.

In summary, the evolution of cohesion is primarily driven by the combined effects of reduced dry unit weight, breakdown of the mineral skeleton, dominance of the soft organic phase, and enhanced plastic deformation. The interval 17%–21% thus constitutes a possible transition interval for the structural degradation and strength loss of organic soil. It is worth noting that the threshold mechanism is further modulated by the water‑binding affinity of hydrophilic organic matter. The threshold mechanism is further modulated by the water‑binding affinity of hydrophilic organic matter. The abundant polar functional groups (e.g., carboxyl, hydroxyl and amine) in sewage‑sludge organics retain substantial bound water through hydrogen bonding and osmotic effects, which reduces interparticle effective stress and consequently lowers shear strength even under constant total stress. In parallel, extracellular polymeric substances (EPS) behave as viscoelastic gels that coat mineral surfaces and form compliant interlayers between adjacent particles. At intermediate organic contents, these EPS films gradually replace rigid mineral‑to‑mineral contacts, facilitating particle reorientation and reducing the overall stiffness – an effect analogous to the “softening” behaviour observed in sludge rheology [39,40]. This combined water‑binding and viscoelastic bridging reinforces the proposed transition from void‑filling to skeleton‑replacement and ultimately to organic‑dominated fabric, providing a more complete physicochemical explanation for the observed strength evolution. As can be seen from Fig 20, for high-liquid-limit organic silt, the internal friction angle varies slightly with organic content and remains stable at a low level of 1–3°. This indicates that organic matter contributes marginally to the friction angle. Instead, it affects the strength of organic soils primarily through the cementation effect on cohesion.

## Conclusion

To investigate the influence of water content on soil strength, organic soils with varying initial moisture content were normalized and tested for UCS. Unconsolidated undrained triaxial tests were performed on organic soils with varying organic contents to evaluate the effect of organic content on strength characteristics. The main conclusions drawn for organic high‑liquid‑limit silt are as follows:

(1) The dry unit weight of organic soils decreases exponentially with normalized moisture content (see Table 3). Similarly, as the organic content increases, the dry unit weight of organic soils decreases following an exponential‑function relationship (see Eq (2)). By contrast, the liquid limit and plastic limit of organic soils increase in a binomial‑function manner with organic content (see Eqs (3) and (4)).
(2) The critical normalized water content *w*_0_/*w*_L_ for the transition from strain-softening to strain-hardening behavior of the four organic soils ranges from 0.45 to 0.55. A power-function decrease in unconfined compressive strength (*q*_u_) with rising normalized moisture content (*w*_0_/*w*_L_) is observed for organic soil, described by the regression equation *q*_u_ = 3.647 (*w*_0_/*w*_L_)^−2.749^ with a correlation coefficient of 0.94.
(3) Under confining pressures of 100, 200, and 300 kPa, and under unconfined conditions (0 kPa), the failure stress decreases with increasing organic content. The organic‑content range of 17–21% may constitute a transition interval for sharp structural degradation in organic soils. The unconfined compressive strength decreases linearly with rising organic content, that is *q*_u_ = −2.090 *w*_u_ + 70.007 with a correlation coefficient of 0.961.
(4) The internal friction angle of organic soils is generally low (1–3°) and shows no significant variation with organic content. Cohesion declines with increasing organic content (*w*_u_): it decreases slowly when (*w*_u_ < 17%), drops sharply at (17% < *w*_u_ < 21%), and stabilizes at about 6 kPa when (*w*_u_ > 21%), beyond which further increases in organic content have little effect on soil strength. Thus, the range 17%–21% constitutes a possible transition interval for the structural degradation and strength loss of organic soil.
(5) Variations in cohesion arise primarily from the combined effects of reduced dry unit weight, disintegration of the mineral particle skeleton, dominance of the soft organic matrix, and enhanced plastic deformation under loading. Organic matter has little effect on the internal friction angle. It predominantly influences the cohesion of organic soils via cementation, thereby affecting soil strength.
